# Gingival Transcriptome of Innate Antimicrobial Factors and the Oral Microbiome With Aging and Periodontitis

**DOI:** 10.3389/froh.2022.817249

**Published:** 2022-03-07

**Authors:** Jeffrey L. Ebersole, Sreenatha Kirakodu, Linh Nguyen, Octavio A. Gonzalez

**Affiliations:** ^1^Department of Biomedical Sciences, School of Dental Medicine, University of Nevada Las Vegas, Las Vegas, NV, United States; ^2^Center for Oral Health Research, College of Dentistry, University of Kentucky, Lexington, KY, United States; ^3^Division of Periodontology, College of Dentistry, University of Kentucky, Lexington, KY, United States

**Keywords:** non-human primate, aging, microbiome, periodontitis, antimicrobial factors

## Abstract

The epithelial barrier at mucosal sites comprises an important mechanical protective feature of innate immunity, and is intimately involved in communicating signals of infection/tissue damage to inflammatory and immune cells in these local environments. A wide array of antimicrobial factors (AMF) exist at mucosal sites and in secretions that contribute to this innate immunity. A non-human primate model of ligature-induced periodontitis was used to explore characteristics of the antimicrobial factor transcriptome (*n* = 114 genes) of gingival biopsies in health, initiation and progression of periodontal lesions, and in samples with clinical resolution. Age effects and relationship of AMF to the dominant members of the oral microbiome were also evaluated. AMF could be stratified into 4 groups with high (*n* = 22), intermediate (*n* = 29), low (*n* = 18) and very low (*n* = 45) expression in healthy adult tissues. A subset of AMF were altered in healthy young, adolescent and aged samples compared with adults (e.g., APP, CCL28, DEFB113, DEFB126, FLG2, PRH1) and were affected across multiple age groups. With disease, a greater number of the AMF genes were affected in the adult and aged samples with skewing toward decreased expression, for example WDC12, PGLYRP3, FLG2, DEFB128, and DEF4A/B, with multiple age groups. Few of the AMF genes showed a >2-fold increase with disease in any age group. Selected AMF exhibited significant positive correlations across the array of AMF that varied in health and disease. In contrast, a rather limited number of the AMF significantly correlated with members of the microbiome; most prominent in healthy samples. These correlated microbes were different in younger and older samples and differed in health, disease and resolution samples. The findings supported effects of age on the expression of AMF genes in healthy gingival tissues showing a relationship to members of the oral microbiome. Furthermore, a dynamic expression of AMF genes was related to the disease process and showed similarities across the age groups, except for low/very low expressed genes that were unaffected in young samples. Targeted assessment of AMF members from this large array may provide insight into differences in disease risk and biomolecules that provide some discernment of early transition to disease.

## Introduction

The oral mucosa is a critical protective interface between external and internal environments and serves as a barrier to the myriad of microbial species in the oral microbiome. Due to the inherent structure where hard tissues (i.e., teeth) break through an intact epithelial barrier, this anatomical region is one where there is a significant risk of bacterially induced infection and inflammation.

To help manage this continual septic environment, a diverse family of antimicrobial factors is produced, which defend the oral cavity and other mucosal surfaces of the body. The oral cavity is home to the second-most diverse microbiome in humans, and the health of the mouth is influenced by the presence of autochthonous bacteria, as well as by intrinsic and extrinsic host factors. Prior to the evolution of adaptive immunity, innate immunity played a role as the principal defense system. The innate immune system augmented the physical and chemical barriers by producing antimicrobial biomolecules (antimicrobial factors; AMF) with functionality against Gram-positive and negative bacteria, parasites, fungi, and viruses [1–3]. Thus, these factors, as a first line of defense, have multiple functions and properties that influence aspects of innate defenses and contribute to regulation of colonization by microorganisms.

The host expresses these protective molecules from immune and non-immune cells that are released into the local environment to act rapidly to combat invasion and infection with bacteria and other microorganisms. Oral epithelial cells, salivary glands and neutrophils secrete a wide profile of antimicrobial gene products detected in gingival crevicular fluid and saliva [2, 4–9]. Many of these AMFs are also highly concentrated in GCF providing a local microenvironment enriched in factors to help control the supra- and subgingival microbiomes [1, 3, 10]. Nevertheless, mechanistic considerations of this system support that the antimicrobial capacity of oral fluids permits and even enhances colonization of the oral cavity by various microorganisms, but prevents extensive microbial overgrowth of the oral and oropharyngeal tissues.

The mechanism of action of these AMFs against microbes and pathogens is attributed to the disruption of the microbial cell membrane [11–13], competition for nutrients [14–16], and microbial aggregation and mechanical or biological removal [17–19]. Also of importance is evidence regarding substantial variation between different species of oral bacteria and even strains of the same species in susceptibility to individual AMFs, such as LL-37 (CAMP) and hBD3 [1, 20–22]. In particular, major pathobionts including *P. gingivalis, A. actinomycetemcomitans* and *F. nucleatum* have shown relative resistance to some of these AMFs [23–26]. Thus, documenting the portfolio of the AMFs that are produced/released under different stimuli, as well as modulating influences (e.g., smoking, diabetes, nutrition etc.) will help in understanding the variation in disease expression, progression, and risk for periodontal diseases [13, 20].

The aim of this study was to map the gingival tissue AMF profile to determine alterations in disease and lesion resolution, as well as the impacts of age on this profile. The hypothesis of this study was that the gingival tissues would present an AMF expression profile with patterns affected by age and with specific features of AMF responses that would be correlated with certain bacteria or clusters/complexes of bacteria.

## Materials and Methods

### Animals and Diet

Rhesus monkeys (*Macaca mulatta*) (*n* = 36; 17 male, 19 female) housed at the Caribbean Primate Research Center at Sabana Seca, Puerto Rico were examined for periodontal health [27–29] and were used to determine the results from a ligature-induced periodontitis model. Young (≤ 3 years); adolescent (3–7 years); adult (12–16 years); and aged (18–23 years) were used in the study with 9 animals/group. The non-human primates were fed a 20% protein, 5% fat, and 10% fiber commercial monkey diet (diet 8773, Teklad NIB primate diet modified: Harlan Teklad, Madison, WI). The diet was supplemented with fruits and vegetables, and water was provided *ad libitum* in an enclosed corral setting.

As we have reported previously the protocol was approved by the Institutional Animal Care and Use Committees (IACUC) of the University of Puerto Rico and University of Kentucky and a ligature disease model was utilized [30]. The clinical examination included probing pocket depth (PPD) and bleeding on probing (BOP; 0–5 scale) [31]. Periodontal health was defined by mean Pocket Depth (PD) ≤ 3.0 mm and mean Bleeding on Probing (BOP) ≤ 1 (0–5 scale) in a full mouth examination excluding 3rd molars and canines [30]. Ligature-induced periodontal disease was initiated as we have previously reported [30] and gingival and subgingival plaque samples taken at 0.5, 1, and 3 months (Initiation/Progression), and 2 months after removal of ligatures and local factors (Resolution). Determination of periodontal disease at the sampled site was documented by assessment of the presence of BOP and probing pocket depth of >4 mm, as we have described previously [28]. Changes in these clinical measures of BOP and PPD in health, during disease initiation and progression, and with resolution in these age groups have been described previously [32]. Briefly, all animals demonstrated significant increases in BOP within 0.5 months, with somewhat elevated levels in the younger age groups. PPD increases were noted in all animals across all age groups with peak disease at 1 or 3 months. However, in both young and adolescent animals, the PPD measures for rate of change and peak levels of disease were less than in the adult and aged group. At resolution, both BOP and PPD measures decreased across all age groups, albeit generally remaining above measures for the baseline, healthy tissues.

### Microbiome Analysis

Subgingival bacterial samples were obtained from the 36 animals by a curette and analyzed using a MiSeq instrument [33, 34] for the total composition of the microbiome from each sample [35, 36]. Sequences were clustered into phylotypes based on their sequence similarity and these binned phylotypes were assigned to their respective taxonomic classification using the Human Oral Microbiome Database (HOMD V13) (http://www.homd.org/index.php?name=seqDownload &file&type=R) as we have described previously [33]. Raw data were deposited at the NIH NCBI (BioProject ID PRJNA516659). Statistical differences of bacterial OTUs were determined with a *t-test* (*p* < 0.05). Correlations of OTUs within the oral microbiome were determined using a Pearson correlation coefficient analysis (*p* < 0.05). Correlations between the microbiome components and the gingival gene expression were determined only for matching samples derived from the same tooth in each of the animals. Matching samples with sufficient microbiome signals were compared for 46 samples in adults and 25 samples from the young group obtained at health and throughout the ligature model. As we have reported previously [33], of 396 OTUs identified in the non-human primate oral samples the targeted OTU selection for this study was 49 in the young/adolescent samples and 58 in the adult/aged samples with a relative abundance coverage of ~88% and ~91% of reads in all samples.

### Gingival Tissue Sample Collection and mRNA Analysis

Gingival tissue samples of healthy and diseased sites were surgically collected and total RNA extracted for microarray analysis. The analytic hybridization was to the GeneChip^®^ Rhesus Gene 1.0 ST Array (Affymetrix, Santa Clara, CA, USA) for the ligature-induced periodontitis model similar to methods we have described previously [27, 28, 37–39].

### Data Analysis

Genes for the array of the innate antimicrobial factors (*n* = 114) targeted in the analysis as identified in Table 1. Beyond the specific Affymetrix probe annotation provided by the company, we annotated within the GeneChip^®^ Rhesus Gene 1.0 ST Array additional probes for the host genes in this microarray. These included unannotated probes, whereby the nucleotide base sequence (https://www.affymetrix.com/analysis/index.affx#1_2) for each probe ID was subjected to a Blast (https://blast.ncbi.nlm.nih.gov/Blast.cgi) query that identified the *M. mulatta* gene ID with the greatest percent identify for the specific sequence. We selected the most targeted gene ID that always showed >90% identity and routinely >95% identity for annotating the gene list for the analysis.

**Table 1:** Affymetrix Rhesus GeneChip 1.0 probes and gene IDs.

**Probe ID**	**Gene ID**	**Product**	**Probe ID**	**Gene ID**	**Product**
13657000	ADM	Adrenomedullin	13607401	CST7	Cystatin 7
13726537	APP	Amyloid beta precursor protein	13607369	CST8	Cystatin 8
13690380	AZU1	Azurocidin 1	13613078	CST9	Cystatin 9
13771401	B2M	Beta-2-macroglobulin	13817566	CST9L	Cystatin 9-like
13611789	BPIFB1	BPI Fold Containing Family B Member 1	13703548	CSTA	Cystatin A
13611309	BPI (CAP57)	Bactericidal Permeability Increasing Protein	13725659	CSTB	Cystatin B
13611807	BPIFA1	BPI Fold Containing Family A Member 1	13607358	CSTL1	Cystatin Like 1
13611830	BPIFA2	BPI Fold Containing Family A Member 2	13783258	CTSG	Cathepsin G
13611813	BPIFA3	BPI Fold Containing Family A Member 3	13664108	CTSL	Cathepsin L
13611823	BPIFA4P/LATH	BPI Fold Containing Family A Member 4	13625511	DCD	Dermcidin
13611885	BPIFB2	BPI Fold Containing Family B Member 2	13790332	DEFA1/MNP1A	Defensin Alpha 1
13611856	BPIFB3	BPI Fold Containing Family B Member 3	13833190	DEFA3	Defensin Alpha 3
13611841	BPIFB4	BPI Fold Containing Family B Member 4	13786263	DEFA4	Defensin Alpha 4
13611870	BPIFB6	BPI Fold Containing Family B Member 6	13786265	MNP2/DEFA5	Defensin Alpha 5
13614378	BPIFC	BPI Fold Containing Family C	13790317	DEFA6	Defensin Alpha 6
13649823	CALCA	Calcitonin Related Polypeptide Alpha	13790306	DEFB1	Defensin Beta 1
13712310	CAMP	Cathelicidin Antimicrobial Peptide	13786259	DEFB4A/B	Defensin Beta 4A/B
13766180	CCL28	C-C Motif Chemokine Ligand 28	13786254	DEFB103A/B	Defensin Beta 103A/B
13777140	CHGA	Chromogranin A	13786233	DEFB104A/B	Defensin Beta 104A/B
13613073	CST11	Cystatin 11	13790341	DEFB105A/B	Defensin Beta 105A/B
13607382	CST2	Cystatin 2	13786229	DEFB106A/B	Defensin Beta 106A/B
13613082	CST3	Cystatin 3	13790338	DEFB107A/B	Defensin Beta 107A/B
13826456	CST4	Cystatin 4	13786278	DEFB108B	Defensin Beta 108B
13613088	CST5	Cystatin 5	13746694	DEFB110	Defensin Beta 110
13654297	CST6	Cystatin 6	13746691	DEFB113	Defensin Beta 113
13746688	DEFB114	Defensin Beta 114	13672583	LPO	Lactoperoxidase
13612123	DEFB115	Defensin Beta 115	13706136	LTF	Lactotransferrin
13606458	DEFB116	Defensin Beta 116	13619325	LYZ	Lysozyme
13612117	DEFB118	Defensin Beta 118	13797016	MBL2	Mannose Binding Lectin 2
13612111	DEFB123	Defensin Beta 123	13756581	MUC7	Mucin 7, Secreted
13606439	DEFB124	Defensin Beta 124	13735015	NPY	Neuropeptide Y
13606465	DEFB126	Defensin Beta 126	13593402	PADI2	Peptidyl Arginine Deiminase 2
13606469	DEFB127	Defensin Beta 127	13581278	PADI4	Peptidyl Arginine Deiminase 4
13612140	DEFB128	Defensin Beta 128	13610899	PI3/SKALP	Peptidase Inhibitor 3
13606475	DEFB129	Defensin Beta 129	13821042	PGLYRP1	Peptidoglycan Recognition Protein 1
13786281	DEFB130A	Defensin Beta 130A	13599233	PGLYRP3	Peptidoglycan Recognition Protein 3
13786284	DEFB131A	Defensin Beta 131A	13599241	PGLYRP4	Peptidoglycan Recognition Protein 4
13786287	DEFB134	Defensin Beta 134	13593639	PLA2G2A	Phospholipase A2 Group IIA
13790347	DEFB135	Defensin Beta 135	13751874	PPBP (CXCL7)	Pro-Platelet Basic Protein
13786290	DEFB136	Defensin Beta 136	13796562	PRF1	Perforin 1
13798980	DMBT1	Deleted In Malignant Brain Tumors 1	13648572	PRG2/BMPG	Proteoglycan 2, Pro Eosinophil Major Basic Protein
13672570	EPX	Eosinophil Peroxidase	13817862	PRH1	Proline Rich Protein HaeIII Subfamily 1
13660411	FCN1	Ficolin 1	13616577	PRH2	Proline Rich Protein HaeIII Subfamily 2
13665140	FCN2	Ficolin 2	13774182	RNASE7	Ribonuclease A Family Member 7
13594266	FCN3	Ficolin 3	13599250	S100A12	S100 Calcium Binding Protein A12
13753483	FGF2	Fibroblast Growth Factor 2	13599267	S100A7	S100 Calcium Binding Protein A7
13599193	FLG2	Filaggrin Family Member 2	13599257	S100A8	S100 Calcium Binding Protein A8
13636114	FN1	Fibronectin 1	13587085	S100A9	S100 Calcium Binding Protein A9
13696432	GALP	Galanin Like Peptide	13801212	SFTPA1	Surfactant Protein A1
13818738	GNLY	Granulysin	13796073	SFTPD	Surfactant Protein D
13693567	HAMP	Hepcidin Antimicrobial Peptide	13605622	SLPI	Secretory Leukocyte Peptidase Inhibitor
13738591	H2AC6	H2A Clustered Histone 6	13611542	SPAG4	Sperm Associated Antigen 4
13744613	HSBC4	H2B Clustered Histone 4	13735408	TAC1	Tachykinin Precursor 1
13713258	HRG	Histidine Rich Glycoprotein	13714939	TF	Transferrin
13611295	LBP	Lipopolysaccharide Binding Protein	13748448	VIP	Vasoactive Intestinal Peptide
13665108	LCN1	Lipocalin 1	13605627	WFDC12	WAP Four-Disulfide Core Domain 12
13762706	LEAP2	Liver Enriched Antimicrobial Peptide 2			

The expression intensities across the samples were estimated using the Robust Multi-array Average (RMA) algorithm with probe-level quintile normalization, as implemented in the Partek Genomics Suite software version 6.6 (Partek, St. Louis, MO). The differential expression was initially compared using one way ANOVA across time points within an age group. For genes that had significant mean differences, two sample *t*-tests were used to investigate differences comparing baseline healthy to disease and resolution samples. Statistical significance was considered by a *p*-value < 0.05 adjusted for the number of correlations tested using a Benjamini-Hochberg procedure. The data have been uploaded into GEO accession GSE180588 (https://www.ncbi.nlm.nih.gov/gds). Correlation analyses were determined using a Pearson Correlation Coefficient with a *p*-value < 0.05 and adjusted for the number of correlations tested. Frequently a qPCR technique is used as a validation step for microarray outcomes. While we did not incorporate this approach into the analysis of this large array of AMF genes, we have previously published the use of qPCR to validate 28 genes from the larger microarray dataset including inflammatory cytokines and chemokines, apoptosis genes, and neuropeptides and receptors, and various transcription factors that demonstrated consistent directional changes, albeit, the magnitude of change was frequently somewhat greater with the qPCR [40–43].

Clustering analysis was conducted that included analysis of 18 AMF genes. A parametric *t*-test (α = 0.05) was used to identify the differentially expressed genes and bacteria at 0.5, 1, 3, and 5 months using baseline expression as control. Those that were differentially expressed, in at least at one of the time points in one or more age groups, were identified and entered into this analysis to examine clustering of both age groups and disease time points. The Principal Components Analysis (PCA) plot was created from results generated with BioVinci software (BioTuring Inc.). Transcript data of the 18 AMF genes that had high variance scores from PCA analysis were averaged across all animals in the same age category. Gene expression data was used to create the unrooted dendrogram by calculating the distance matrix with the Euclidean method followed by ward D2 linkage clustering method using the Ape package in R version 4.1.1. The data was normalized on a scale of 0 to 1, with the lowest value set at 0 and the highest value at 1, to generate the heatmap and rooted dendrogram for the cluster analyses. The agglomerative hierarchal cluster analyses were performed by calculating the distance matrix using the Squared Euclidean method and by average linkage (between-group) clustering method using SPSS v27 (IBM).

## Results

### Age Effects on Innate Antimicrobial Factor Gene Expression in Healthy Gingival Tissues

**Figure 1:**
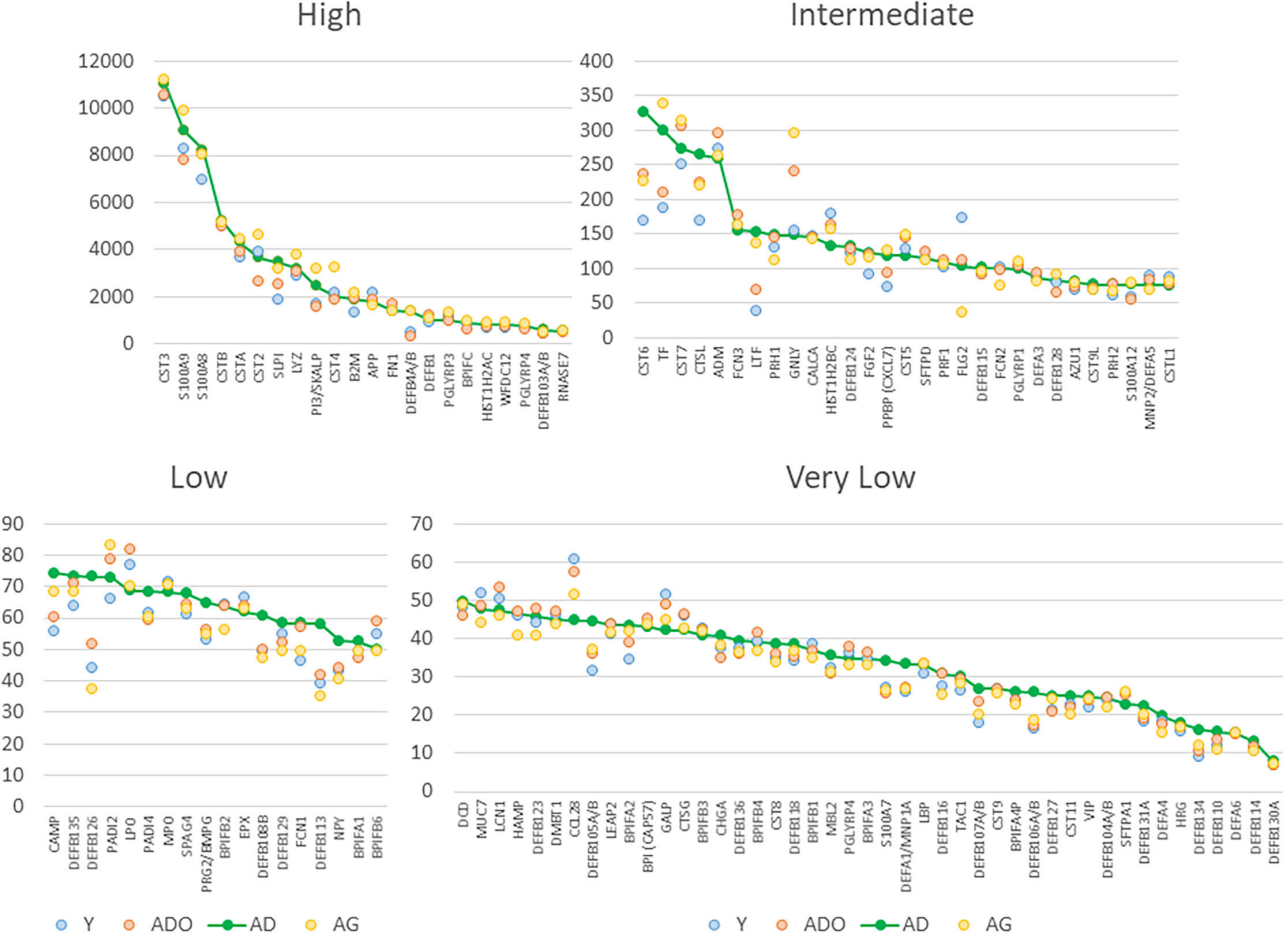
Normalized expression values for antimicrobial factors categorized as high, intermediate, low and very low expression levels based upon the adult healthy tissues. Each point denotes mean value from 9 animals/group. The genes are sequenced based upon the expression level in adult samples.

**Figure 2:**
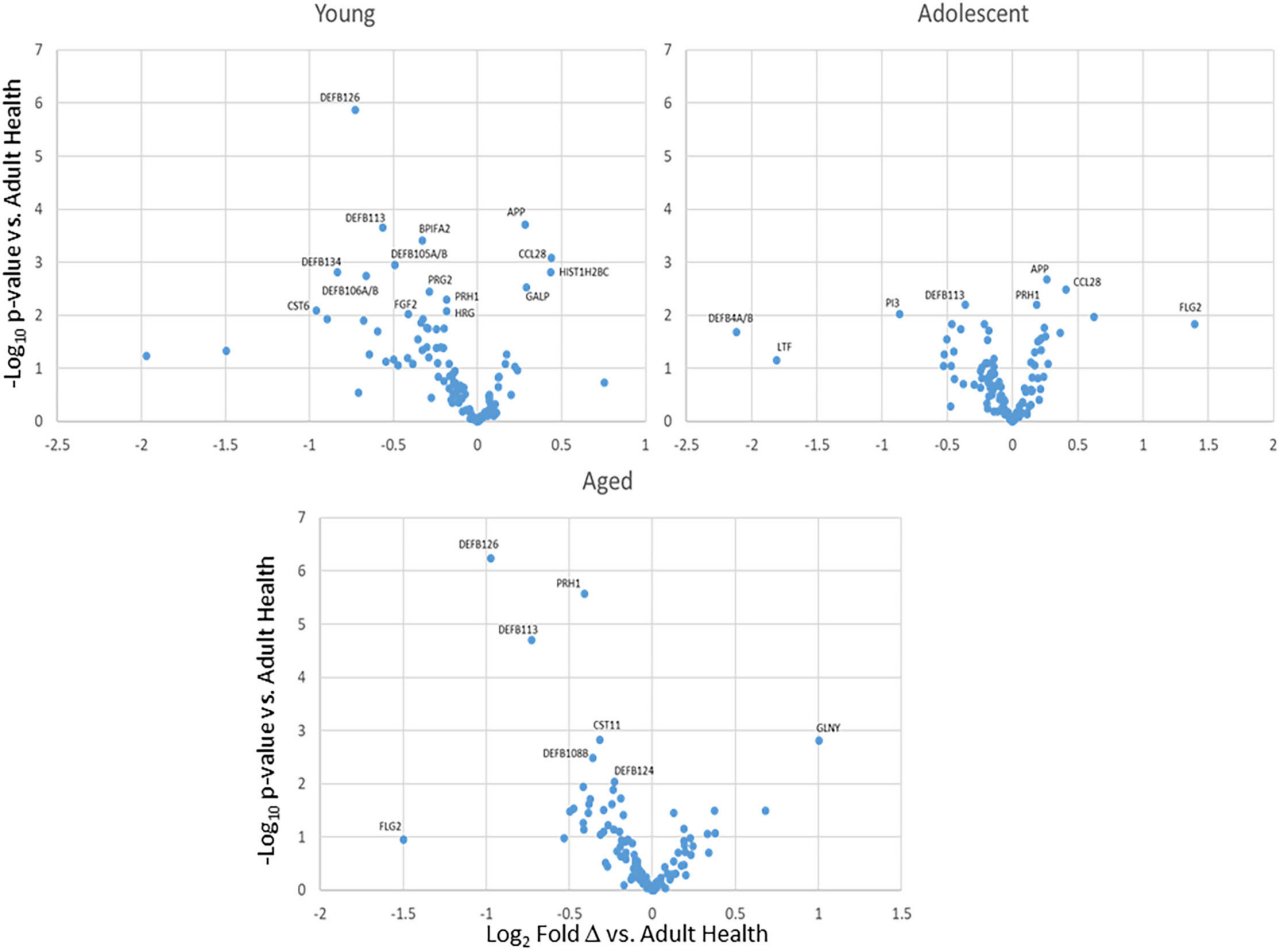
Volcano plots of differential AMF gene expression levels in health tissues from various age groups compared to healthy adults. Each point represent the mean value from 9 animals at baseline.

The normalized expression of transcripts for the 114 innate antimicrobial factors is demonstrated in Figure 1 with the genes stratified into high (*n* = 22), intermediate (*n* =29), low (*n* = 18), and very low (*n* = 45) expression based upon the healthy/baseline adult gingival specimens. In healthy/baseline gingival tissues, generally there were some observed differences in expression based upon age, including variations in both younger and aged animal samples. For example, DEFB4A/B, LTF, and TF were all decreased in the younger age groups, while CST6 (cystatin E/M), DEFB126, DEFB105/A/B, S100A7, and DEFB106A/B were lower in all age groups compared to adult levels. Figure 2 displays volcano plots of the AMF genes that were altered in young, adolescent, and aged samples compared to the adult levels in health. Seventeen genes were 2-fold and/or significantly different (*p* < 0.01) in the young animals. Fewer gene differences were seen in adolescent samples (*n* = 8), while 8 of the genes differed in the aged animals. Of note, APP, CCL28, DEFB113, DEFB126, FLG2, and PRH1 were differentially expressed in healthy gingival tissues across multiple age groups.

### Age Effects on Innate Antimicrobial Factor Gene Expression in Progressing Periodontitis

Supplementary Figures 1A–D depict the alterations in transcript levels for the array of antimicrobial factor genes comparing baseline healthy levels to initiation (0.5 months), progression (1 and 3 months) and resolution (5 months) of periodontal lesions. Examination of fold differences showed a limited number of changes in the highly expressed genes in the young and adolescent animals. A greater number of these genes were affected in the adult and aged samples with skewing toward decreased expression of these genes across all age groups. WDC12, PGLYRP3, and DEF4A/B were decreased in disease across multiple age groups. Supplementary Figure 1B shows the differential expression of the intermediate expressed transcripts for these factors. Consistent findings were decreased FLG2, DEFB128, and increased levels of CTSL and LTF with disease. Low expression genes are shown in Supplementary Figure 1C with no differences in any of the genes in young animals. PADI4 and FCN1 were increased in the other groups, and DEFB126, DEFB113, DEFB106A/B, DEFB110, DEFB127, and DEFA6, were all decreased in the adult samples. Supplementary Figure 1D summarizes the differences in very low expression AMF genes compared to baseline healthy levels in the age groups. Minimal differences were noted in adolescent, adult and aged samples. A number of the AMF appeared decreased in the young samples with disease, albeit, these did not attain a 2-fold difference from baseline expression levels.

**Figure 3:**
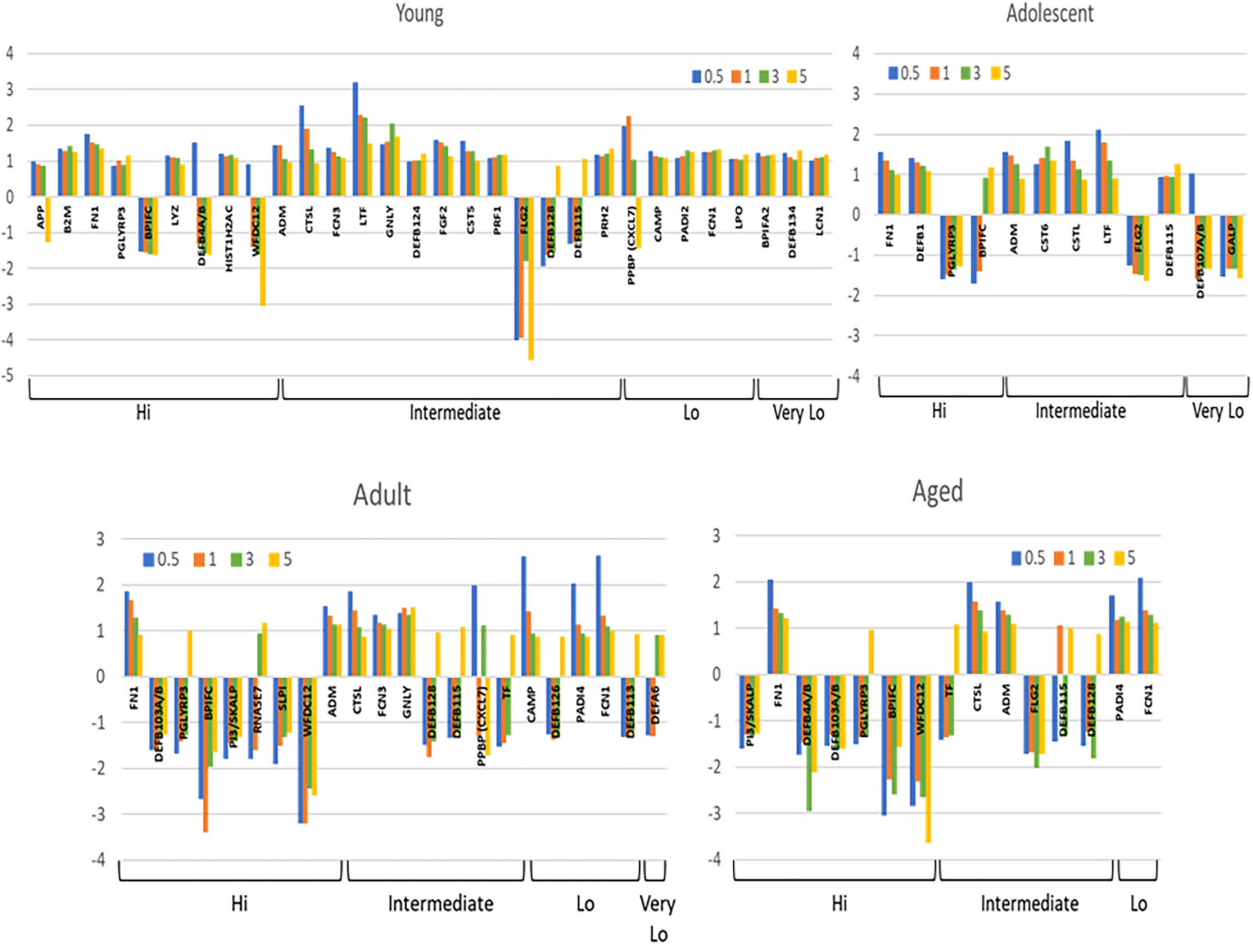
Array of AMF genes that were significantly (*p* < 0.05) differentially expressed at least at one time point during disease and resolution in tissues from the 4 age groups. Bars denote mean fold-difference from baseline at each time point. Genes are organized into expression level categories based upon Figure 1.

Figure 3 summarizes the antimicrobial factor genes that were significantly altered in the different age groups with disease and resolution. The data demonstrate 30 (young) and 12 (adolescent) AMF genes that were increased with FN1, PGLYRP3, ADM, CTSL, LTF, FLG2, and DEFB115 being represented in both these age groups. Of these AMF genes, BPIFC, FLG2, and multiple DEFB genes were decreased in expression with disease in one or both young and adolescent animals. A similar evaluation of these differences in the adult and aged animals is also displayed. First, the number of the affected transcripts was only about 40% of those in the youngest animals. Secondly, about 60% of these genes were significantly decreased in both age groups. Increased FN1, CTSL, ADM, PADI4, and FCN1 was noted in the adult and the aged groups, as well as most being elevated in the younger animal groups. Decreased levels of expression of multiple genes including DEFB4A/B, PGLYRP3, BPIFC, WFDC12, PI3/SKALP, DEFB128 were observed in adults and aged samples, with many of these also decreased in the young and adolescent samples during disease. As the repertoire of genes selected for this study were primarily related to epithelial cell biology, the distribution of the decreased AMF in the older groups versus young samples inferred that the more destructive disease process in the adult and aged animals was reflected in the apparent disruption of the breadth of AMF responses designed to maintain homeostasis.

**Figure 4:**
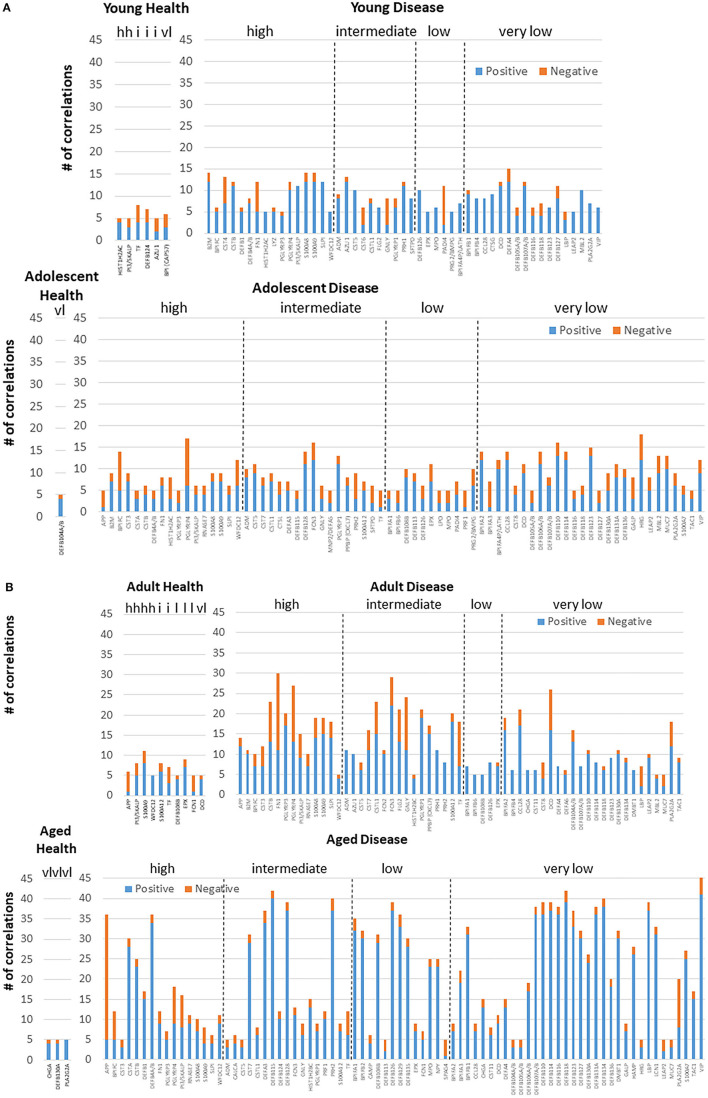
Correlations among the AMF gene in **(A)** young and adolescent health and disease and **(B)** adult and aged health and disease. The bars denote the number of either positive or negative significant correlations for each of the genes showing 5 or more significant correlations across the entirety of the 114 AMF group examined. Genes are organized into expression level categories based upon Figure 1 (h, high; i, intermediate; l, low; vl, very low).

Figures 4A,B examined the concept that there could exist coincident programming and responses across these AMF genes in health and disease. In each case, a rather limited number of correlations across this array of AMF were noted in healthy tissue in any age group, suggesting a rather independent control of these responses to the local microbiome during health. In contrast, there were many more extensive correlations in gene expression across the AMF factors during disease. Interestingly, the frequency of these correlations increased from young through aged animals in the disease samples. Additionally, the pattern of these relationships was striking in the aged animals with a large frequency of significant positive correlations across a wide range of the AMF.

**Figure 5:**
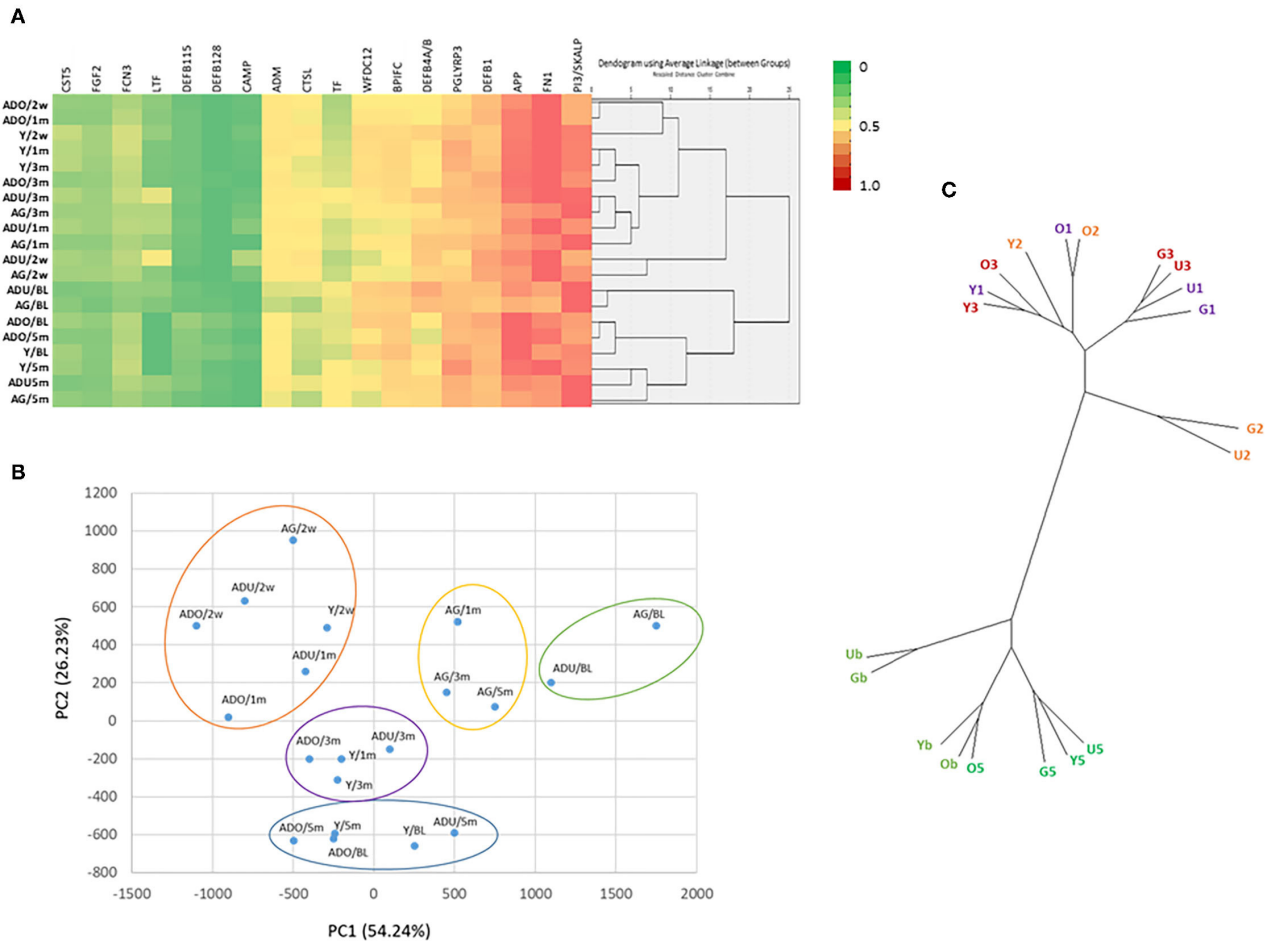
**(A)** Heatmap and clustering using 18 AMF genes that discriminated age and the disease process. **(B)** Principal Components Analysis of 18 AMF genes. Y, young; O, Adolescent; U, Adult; G, Aged samples; b, baseline; 2, initiation; 1 and 3, progression; 5, resolution of disease. Each point denotes the mean of the age group and time point. **(C)** Unrooted tree representation of grouping of the AMF gene expression. Labels are as in **(B)**.

Figure 5 provides a visualization of the relationship among the various AMFs related to both age and health/disease of the gingival tissues. The emphasis of the cluster analysis and heatmap is for 18 of the AMF genes that showed distinct expression differences across the age groups and related to the stage of health, disease, and resolution of the gingival tissues. This analysis identified 5 clusters with the primary grouping based upon stage of disease, with age as a secondary feature. Additionally, similarities in expression were noted between baseline and resolution specimens. The figure also provides a Principal Components Analysis using the same set of genes. This analytic approach also identified 5 groups of expression and also demonstrated disease as the primary feature controlling the AMF expression patterns. Finally, Figure 5 also summarizes these same linkages in an unrooted tree structure. Features show the profiles of AMF expression are quite similar in healthy and resolution samples, albeit the health samples from adult and aged animals were somewhat unique. Secondly, the disease samples in the young and adolescent animals were all in the same general root structure, while progressing disease (i.e., 1 and 3 month) for the adults and aged animals grouped together. Finally, the disease initiation AMF profiles (i.e., 2 weeks) in the adult and aged samples showed some unique features compared to all other disease specimens.

**Figure 6:**
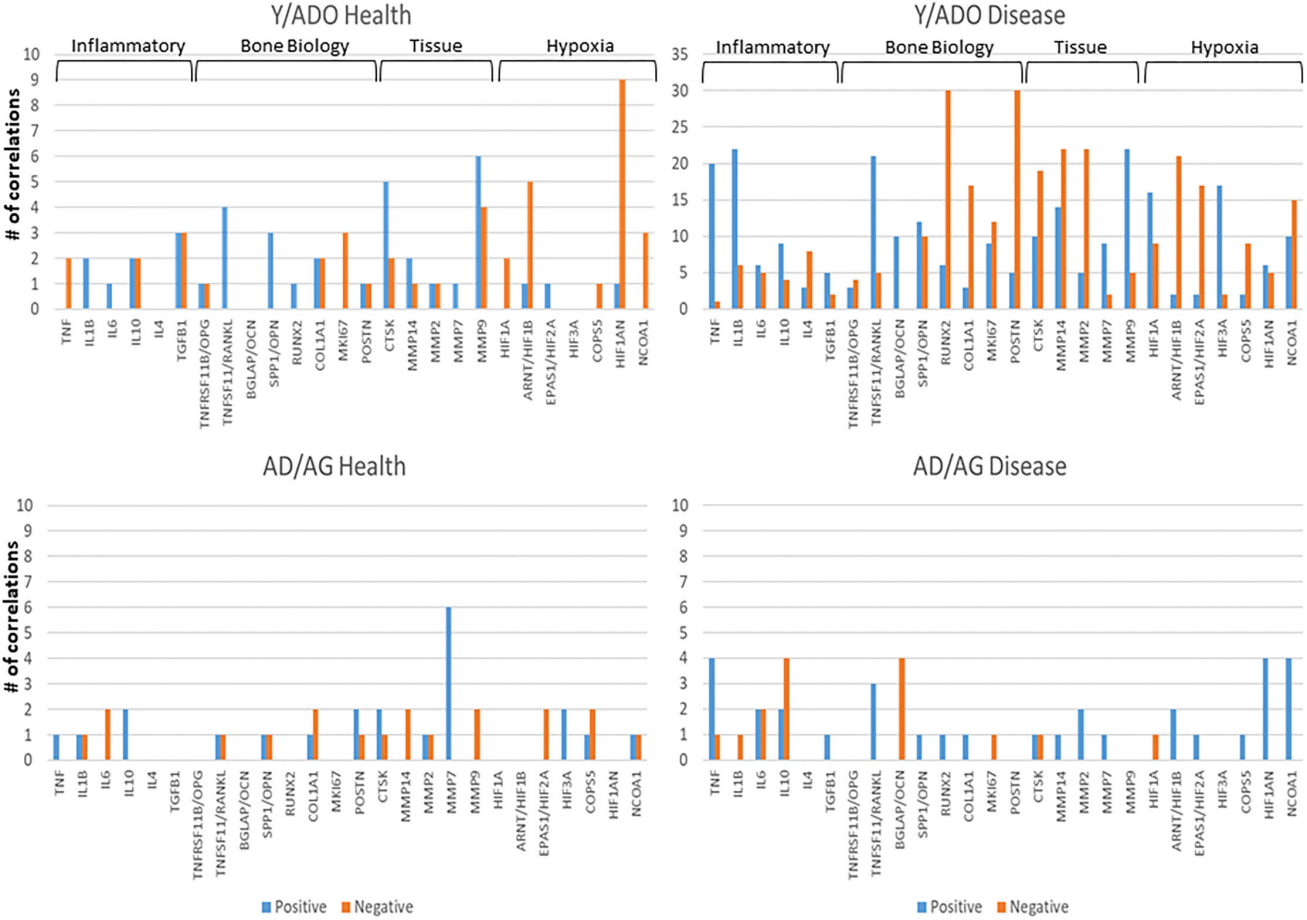
Correlations between AMF gene expression levels and various groups of genes related to the tissue microenvironment in younger (Y/ADO) and older (AD/AG) animal gingival tissues. Colors denote number of significant positive or negative correlations of each tissue gene with the array of AMF genes.

While oral bacteria clearly trigger the destructive inflammatory responses of periodontitis, substantial literature has identified an array of host factors that could directly account for the features of inflammation, soft tissue destruction, and alveolar bone resorption in this disease. As such, we asked the question regarding the relationship between the expression of AMF genes and targeted host genes associated with the local environmental tissue changes in the periodontium. Figure 6 presents the data identifying the frequency of significant correlations in AMF and the genes that could impact the environment and tissues. The striking feature was the much higher frequency of these relationships in the younger animals compared to the older adult/aged group in both health and disease. Also apparent was the large number of these correlations in the younger groups during disease.

### Oral Microbiome Components and Innate Antimicrobial Factor Gene Expression in Health and Disease

**Figure 7:**
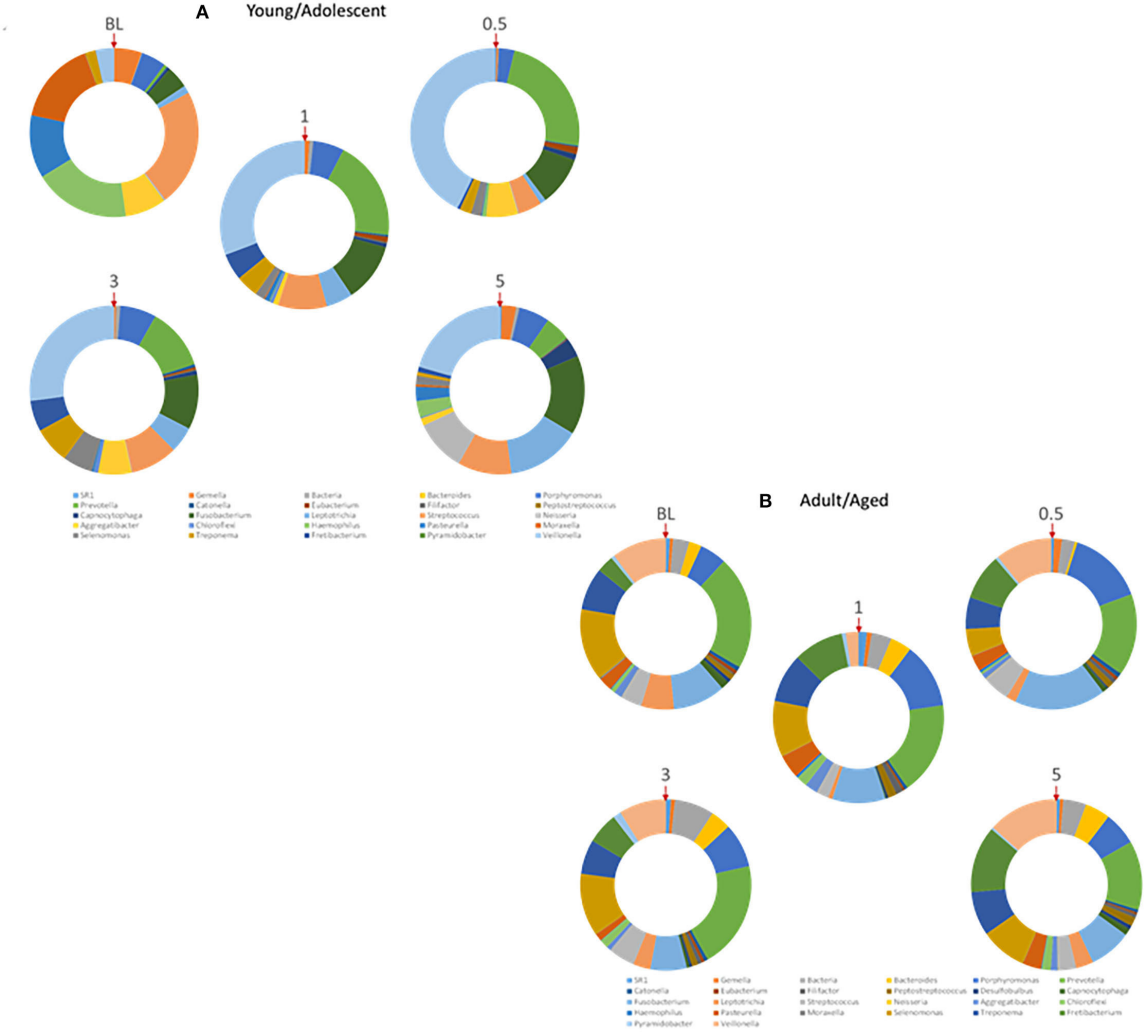
Relative abundance of various genera of bacteria within the microbiomes of **(A)** younger (Young/Adolescent) or **(B)** older (Adult/Aged) gingival tissues. Each ring represents one sampling time point. Red arrow denotes starting point for identification of individual genera.

The oral microbiome in humans and non-human primates is quite complex, with many similarities in the distribution of phyla, genera, and species [44–46]. As it is clear that an array of host factors, including the AMFs interact with components of the microbiome, we examined details of these potential relationships. Figure 7A displays the distribution of the various primary genera of bacteria within the oral microbiome of samples from the younger animals. Substantial changes occur with disease initiation (0.5 months) that are generally retained during disease progression and remain quite distinct from the healthy microbiome even with clinical resolution of disease (5 months). A similar profile of genera is provided in Figure 7B for the older group of animals. While there were many similarities in the microbial distribution during different stages of disease, a striking change was the increase in *Porphyromonas, Fusobacterium*, and *Fretibacterium* with disease in the older animal samples.

**Figure 8:**
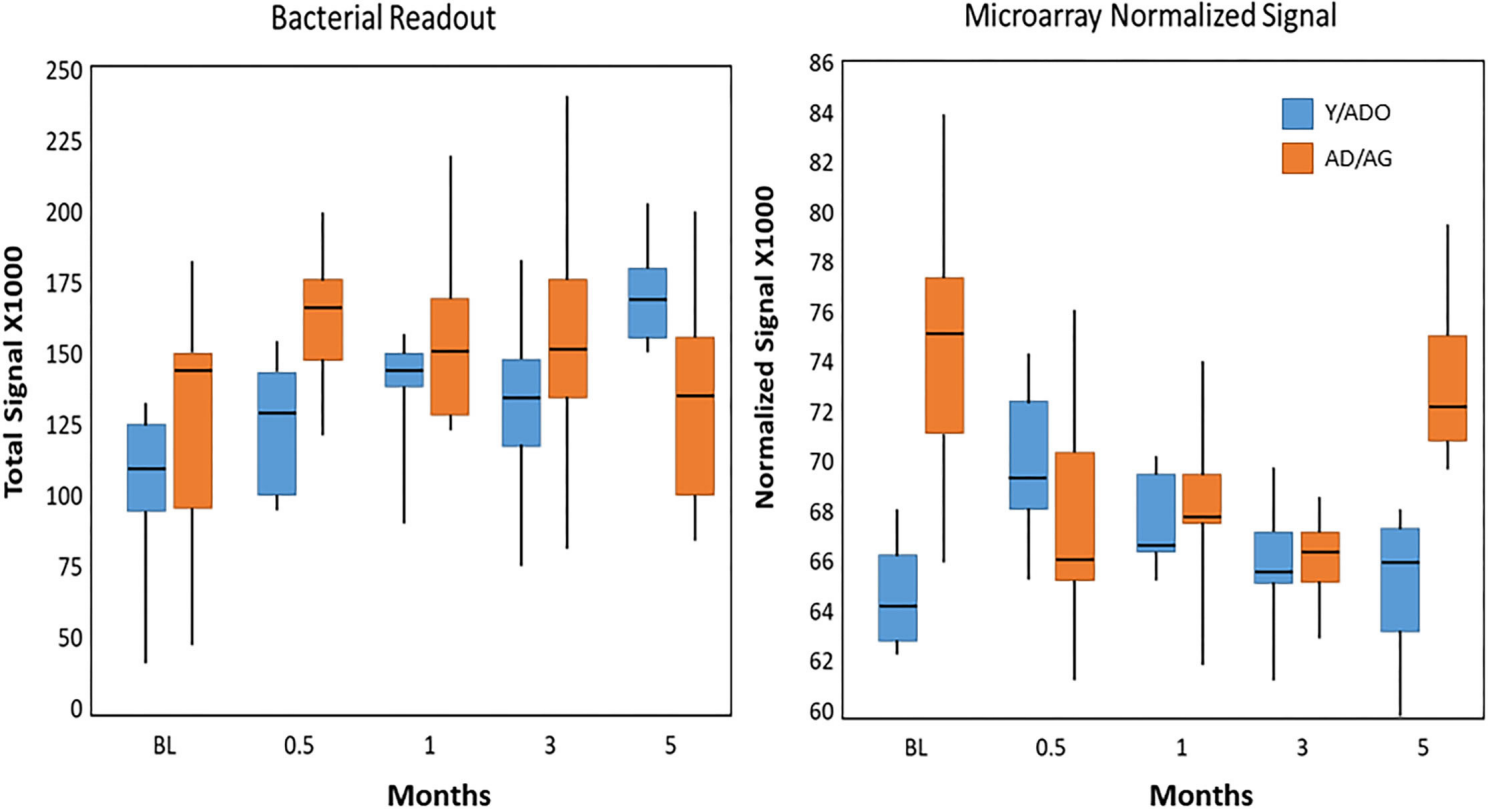
Box and whisker plot of total abundance of the bacterial signals in samples from younger and older animal groups at baseline, with disease, and in resolution samples. Similar plot tabulated the sum of the microarray normalized signals for all AMFs in each sample.

Figure 8 demonstrates the overall relationship between the microbial burden (total bacterial signal readout) and the normalized signal for the microarray values for the entire set of AMFs. The results showed the microbial burden increased with disease in both age groups; however, the levels returned to near baseline in the older group of animals (AD/AG), while remaining increased by nearly 60% in the Y/ADO group. However, the pattern of general AMF gene expression was quite different in the 2 age groups. The levels at baseline were significantly greater in the AD/AG group, but decreased with disease initiation and progression. In the Y/ADO group, the AMF levels increased at the early stages of disease. Both age groups showed AMF gene levels approximating baseline values in the resolution samples.

**Figure 9:**
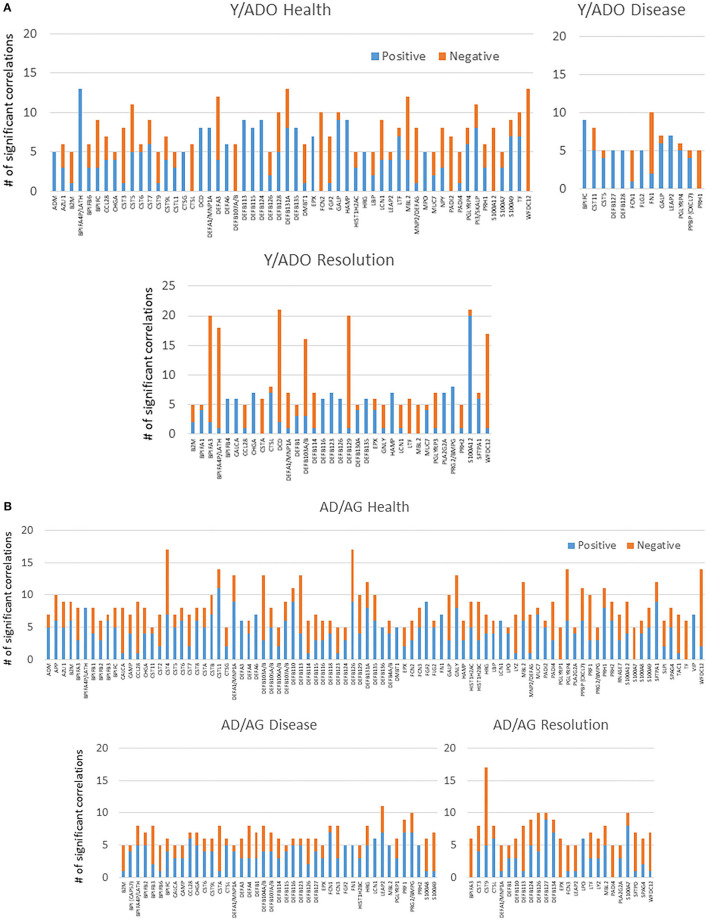
Significant positive or negative correlations between relative abundance of the 49 OTUs **(A)** in the younger group or 58 OTUs **(B)** in the older group included in the microbiome analysis and individual AMF genes. Bars denote number of OTUs correlated with the specific AMF gene. Only AMF genes with significant correlations with 5 or more of the microbial OTUs are included.

Figure 9 shows the relationship between the AMF gene expression levels and correlations with the array of OTUs in the microbiome samples. We included 49 OTUs that comprised readouts accounting for 77–86% in the younger group and 58 OTUs representing 74–82% in the older group. In healthy samples from the younger group (Figure 9A), we identified that CST5, DEFA3, DEFB128, DEFB131A, GALP, MBL2, TF, WFDC12, BPIFA4P, and PI3/SKALP showed elevated frequencies of significant correlations. The frequency of AMF-microbial relationships decreased substantially during disease with only FN1 represented. In the resolution samples, while fewer transcripts were significantly correlated, DCD, DEFB103A/B, DEFB129, S100A12, WFDC12, BPIFA3, and BPIFA4P all showed extensive correlations across the various OTUs. Figure 9B provides a comparison with the adult/aged group samples with 21 genes demonstrating frequent correlations (>10) in the healthy tissues samples. As noted in the younger samples, this was decreased to only PRG2 and LEAP2 in the disease samples. A contrast with healthy tissues was observed in the resolution samples from the adult/aged group, with a limited number of AMF genes showing any correlations with the microbiome components, and only CST9, DEFB126, DEFB127, and S100A7 demonstrating an elevated number of correlations.

**Figure 10:**
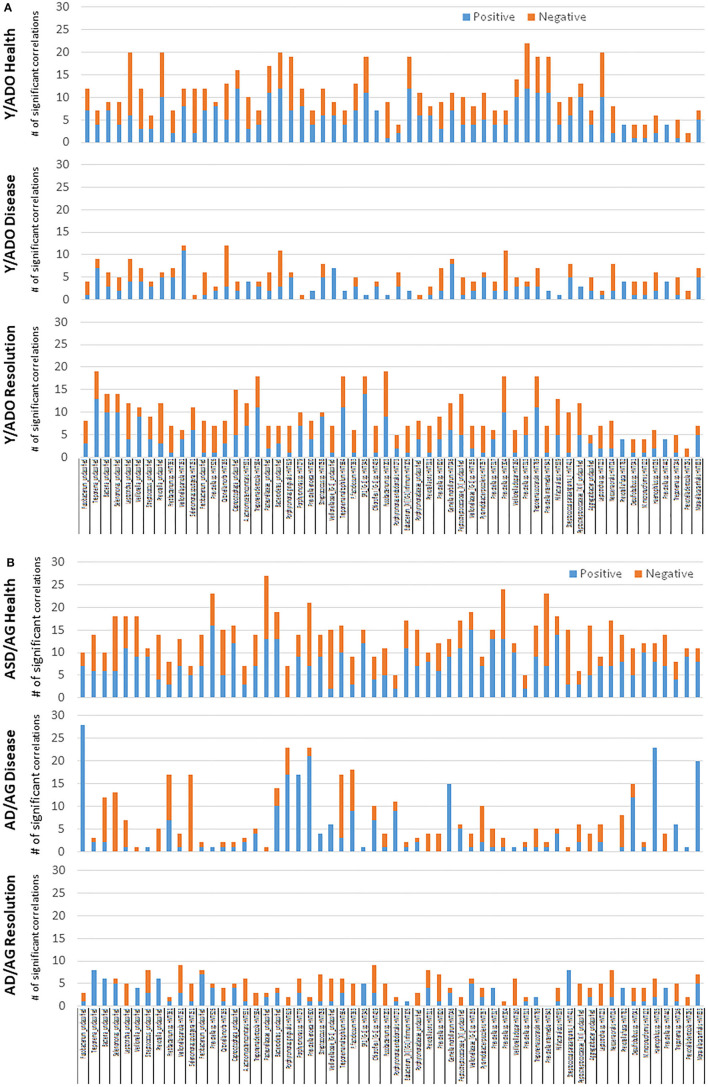
Significant positive or negative correlations between individual AMF genes and relative abundance of specific OTUs **(A)** in the younger group or **(B)** in the older group. Bars denote number of AMF correlated with the specific OTU.

The data was also evaluated to identify the members of the oral microbiome that dominated in correlations with the array of AMF gene expression patterns in the juxtaposed gingival tissues. Figure 10A shows multiple OTUs that were highly correlated with the AMF in healthy young/adolescent samples that included *Leptotrichia*_unclassified, *Prevotella*_unclassified, *Capnocytophaga*_unclassified, *Bacteroidetes*_unclassified, *P. gingivalis* HMT619, SR1_[G-1] sp. HMT345, *E. infirmum* HMT105, *Prevotella* sp. HMT313, *T. socranskii* HMT769, *P. intermedia* HMT643, and *Leptotrichia* sp. HMT223. In disease, these correlations were much more limited with none of the bacteria demonstrating >15 correlations. In contrast, in the resolution samples, 8 OTUs showed an elevated frequency of correlations with the AMF gene expression levels and included *Treponema*_unclassified, *Capnocytophaga*_unclassified, *T. denticola* HMT584, SR1_[G-1] sp. HMT345, *Fusobacterium* sp. HMT203, *Prevotella* sp. HMT304, and *T. socranskii* HMT769.

Figure 10B summarizes the microbial components with numerous AMF correlations in the adult/aged group samples. In the healthy samples, 24 OTUs showed a large number of significant correlations to the AMF transcripts. A different subset of these were seen in disease, highlighted by *Fusobacterium*_unclassified, *Fretibacterium* sp. HMT361, *S. sputigena* HMT151, *P. gingivalis* HMT619, *Porphyromonas* sp. HMT279, *P. enoeca* HMT600, *T. maltophilum* HMT664, *F. fastidiosum* HMT363, *G. morbillorum* HMT046, *Desulfobulbus* sp. HMT041, *Haemophilus* sp. HMT035, and *M. catarrhalis* HMT833. Finally, in the resolution samples, this magnitude of correlations was generally absent in the adult/aged samples.

Supplementary Figure 2 presents a summary of the AMF genes that are most greatly affected by components of the microbiome. In this analysis, the bacterial families that predominated in the microbiome were stratified into high and low levels based upon median family level in the adult/aged samples. We then determined the AMF gene levels based upon the ratio of high/low levels of the microbial families. Most notable was that across the bacteria, the majority of the AMF were increased with lower relative abundance of the various bacterial families. In particular, expression of WFDC12, PBPP, CST5, BPIFC, DEFB103A, PI3/SKALP, SLPI, LTF, and DEF4A/B were most broadly impacted by showing decreased levels with higher levels of multiple families of bacteria in the microbiome. Additionally, *Lactobacillales, Pasteurellales, Selenomonadales*, and *Veillonellales* demonstrated the greatest frequency of lower expression with the higher relative abundance in the samples.

### Microbial Complexes and Inflammatory Gene Expression in Aging

**Figure 11:**
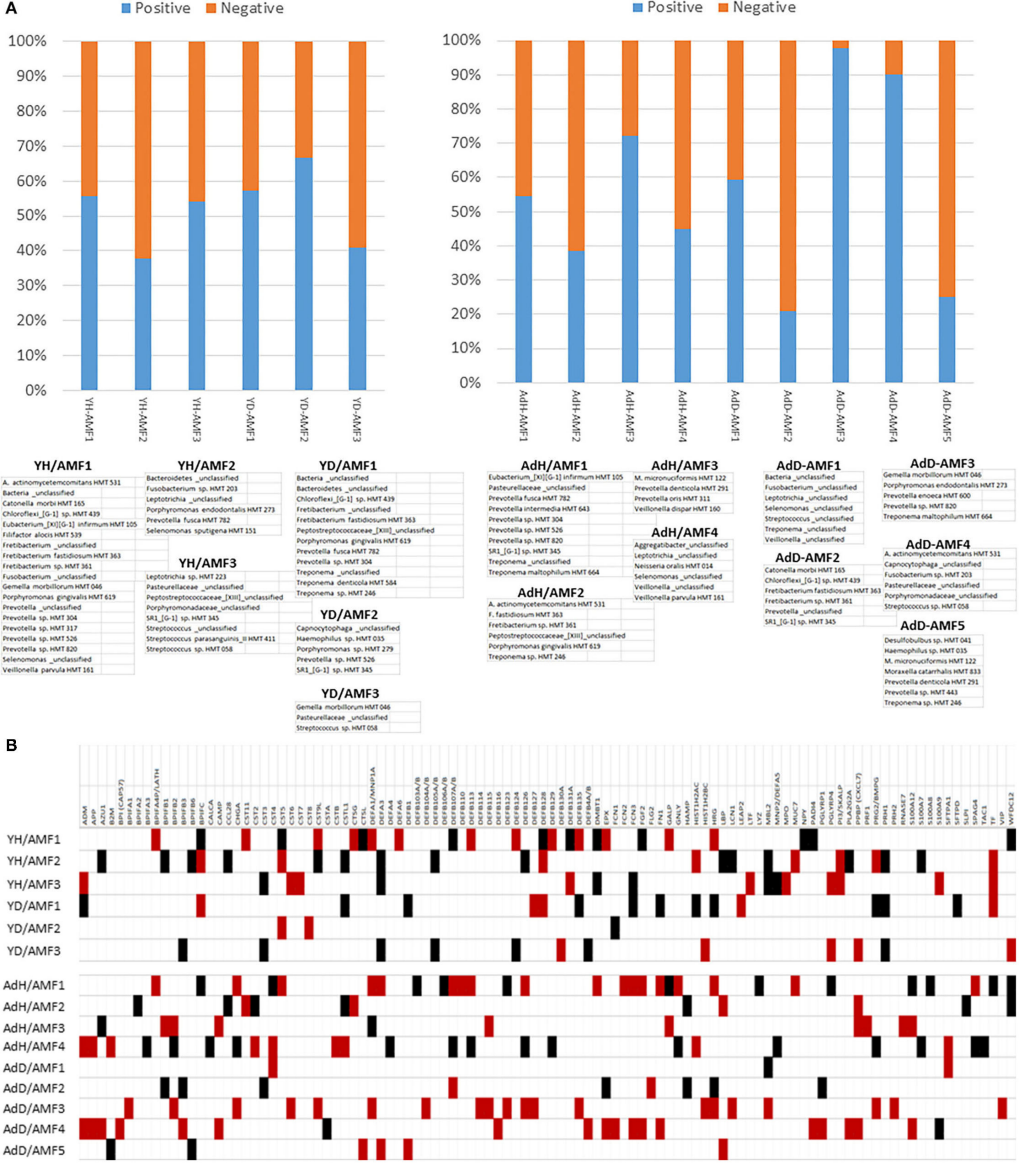
**(A)** Relative distribution of positive and negative correlations across the AMF genes related to specific bacterial complexes in young (Y) or older (Ad) animals samples. Complexes are identified in healthy samples (e.g., YH, AdH) or diseased samples (e.g., YD, AdD). **(B)** Correlations between microbiome complexes and individual AMF gene levels. The red box denotes significant positive correlations and the black denotes significant negative correlations with the array of bacteria in the identified complex.

A pattern of microbiome members and specific groups of the AMF were noted within these individual correlation analyses. This pointed to a feature, in which there appeared complexes of the bacteria that related to a particular profile of the AMF, potentially related to their general or specific antimicrobial features. Figure 11A provides a profile of the characteristics of these complexes related to patterns of AMF expression. First of note was that two bacterial complexes in healthy younger samples primarily showed positive correlations with the AMF, while YH/AMF2 was skewed toward negative correlations with the AMF. The complexes were represented by unique individual microbiome features, with the members comprised of a mix of species that are classically considered more pathogenic and others identified as commensal. With disease, the members of the complexes changed with YD/AMF1 and YD/AMF2 oriented more toward pathogenic species and YD/AMF3 generally considered commensals. This difference was noted with the relative correlation distribution of the complexes to the AMF.

In the adult/aged samples, AdH/AMF1 and AdH/AMF3 showed more dominant positive correlations with the AMF. Of interest was that AdH/AMF1 demonstrated a prevalence of what would be considered pathogenic species, while AdH/AMF3 was generally commensals. In contrast AdH/AMF2 and AdH/AMF4 showed dominant negative correlations with AdH/AMF2 showing mostly more pathogenic species. Substantial changes were observed in the complexes in the adult/aged disease samples. AdD/AMF2 and AdD/AMF5 showed a high prevalence of negative correlations with the AMF and AdD/AMF2 was highly represented by more pathogenic species. In contrast, AdD/AMF3 showed almost entirely significant positive correlations with the AMF, and was composed of species generally considered as commensals.

Figure 11B provides a tabulation of the individual AMF that displayed the predominance of correlations to the various bacterial complexes. First, 103 of the AMF showed significant correlations to one or more of the bacterial complexes. Secondly, specific complexes demonstrated enhanced numbers of these correlations including YH/AMF1, YH/AMF2, AdH/AMF1, AdH/AMF4, AdD/AMF3, and AdD/AMF4. Within the young healthy samples only 10–15% of the individual AMF actually overlapped between the two complexes and all were in an opposite correlation direction. Fewer specific correlations were noted in the young disease samples with unique AMF patterns for each complex and with only BPIFC, CST5, DEFA3, DEFB128, PRH1, and TF showing an elevated frequency of correlations.

The four complexes in the adult/aged samples also showed low overlap of AMF (1–8 AMF similar), while the disease samples exhibited even less overlap (1–3 AMF similar). The AMF genes that demonstrated the greatest prevalence of correlations in these older animal samples included BPIFB2, CHGA, CST4, DEFA1, DEFB107A, DEFB126, HRG, PPBP, and S100A12.

## Discussion

Antimicrobial factors (AMFs) in the oral cavity range from the relatively large agglutinating mucins to ion scavenging molecules, to smaller membrane-perforating cationic peptides [2, 13, 47, 48]. These AMFs are important contributors to maintaining the balance between health and disease in this complex environment. These AMFs are part of the host innate immune response in this environment and are produced by epithelial cells, polymorphonuclear leukocytes, and salivary gland cells to contribute to protection of the oral cavity with overlapping and unique functions. Beyond the direct bacterial effects including affecting microbial viability, and functions of bacterial LPSs and peptidoglycans, many AMFs possess host cell regulatory functions including apoptosis, proinflammatory, and chemotactic activities. Various studies have identified alterations in AMFs in the oral cavity of humans with periodontitis [49–55], as well as demonstrating the capacity of various oral bacteria to trigger production of these biomolecules from various cells in culture. However, generally these studies have focused on one or a few of the innate immune components related to health and disease [9]. This study mapped the relative abundance of 114 AMF genes in non-human primate gingival tissues in health, disease and resolution, as well as the effect of age on this expression. Moreover, this expression was examined related to the presence of the major 50–60 bacterial OTUs in the oral microbiome, and the relationship to genes expressed in the tissues that indicate local environmental features in the gingiva.

Of the AMF characterized in the study, expression levels in healthy tissues demonstrated various AMFs expressed at high to very low levels. Comparing the expression across age showed a limited number of these genes differed, albeit, some unique features were displayed in both young and aged animals, e.g., CST6, CTSL, CAMP, DEFB126, CCL28. Additionally, the number of the AMF genes that differed significantly from adult levels were routinely greatest in samples from young animals, with an overlap of about 50% of the differentially expressed genes across the age groups. Extension of the determination of the AMF expression focused on changes related to disease initiation and progression, as well as in tissues with clinically resolved lesions. Again, nearly twice as many of the AMFs were altered in the young samples vs. the other age groups. While most of these differences were identified in disease samples, a subset of the AMF remained altered even in the resolution samples. Also, in the young and adolescent groups 60–70% of the genes were upregulated during disease, while nearly? were down-regulated in the adult and aged animals. This observation could be related to a more resilient oral microbiome that we described before in aging vs. young animals. As such, the young animals appeared to show more significant dysbiotic changes with the disease process. Nevertheless, there was extensive overlap in the targeted AMF genes that were affected in all age groups.

The detection of overlapping AMF expression features within the samples across age groups suggested the potential for distinctive correlation patterns of AMF. Exploring these correlations in healthy tissues showed a somewhat limited frequency of significant correlation across the array of AMFs. Young and adolescent animals displayed ~20% of the AMF genes with numerous correlations, while 30% of the genes showed high correlations in the adults and nearly 50% of the genes were highly correlated in the aged samples. This was somewhat unexpected, as both innate and adaptive immune responses are generally negatively associated with the aging process. However, it appears that many of the AMFs may be conserved during aging, and show an increased similarity in regulation of expression. Also, of note in this dataset was that previous reports indicated that the antimicrobial factor changes in saliva did not appear to cluster around similar functions for the biomolecules, but showed diverse properties including LPS-binding protein (LBP) altered coupled with cystatins, lipocalin 1, and perforin 1 [56, 57]. Here the array of altered AMFs could be assigned to multiple functional targets and may more broadly reflect changes in the microbiome, to which various host cells are responding. A striking feature of the AMF gene expression patterns was exposed when examining the clustering of the AMF expression in gingival tissues from the various age groups, and in health and disease. Our previous results have supported that the transition from health to disease was a major driving feature of altered gingival gene expression patterns for multiple pathways [27, 30, 37, 38, 40, 41, 58–62]. While some age differences were observed, the major changes appeared to be disease related. In a similar fashion with the AMF, cluster analysis clearly emphasized that the characteristics of the gingival tissues (i.e., health, disease, resolution) appeared to be the major controlling factor in the relationship of AMF expression. This suggested that evolution of the AMF producing pathways was similar with aging processes. These biomolecules are a downstream expression of numerous molecular pathways of innate immunity. Thus, some common features of cell receptors, cellular signaling, or transcription and translation processes may be under similar control in aging, more than previously considered. Identification of these regulatory components should provide improved insight into alterations in host resistance to infection, neoplasia, and even autoimmunity that could occur with aging. As noted since the AMFs represent an array of biomolecules with various cellular origins within the gingival tissues, an understanding of the temporal cellular differences during disease initiation and resolution would add additional knowledge to the features of the specific cellular responses that occur and result in alter AMF levels. Current technology using single cell RNASeq studies provides a method to explore the cellular composition in complex tissues and uses specific mRNA patterns to describe the tissue composition [63]. Related to this, various approaches have been used to explore existing large gene expression datasets to also document the character of the cellular makeup of tissues. The approach uses cell-specific convolution and a computation method of population-specific expression analysis (PSEA) to describe the cell composition [64–66]. Specifically, Momen-Heravi et al. [67] has recently reported on the use of this technique to characterize the cellular composition of human gingival tissues in health and disease. The AMF data was extracted from a larger microarray study that is amenable to this type of bioinformatics exploration and should add insights into the changes in cellular composition that underpin these AMF gene alterations in disease. An additional focus can also be generated from the breadth of findings in this study such that hypothesis generation can be developed based upon the more detailed findings of individual AMF and their potential molecular role in homeostasis and/or disease. An example of this concept is in the findings with ficolin (FCN1) that was generally significantly positively related to the disease process. The ficolins are soluble pattern recognition molecules that contribute to complement activation via the lectin pathway [68]. Ficolins in serum are complexed with MBL-associated serine proteases and bind to carbohydrates present on the surface of microbes as components of innate immune responses. Related to periodontitis, studies have shown that LPS-induced inflammation can induce a significant ficolin response [69]. While the ficolin response/function has very limited findings in periodontitis studies have shown that the mirolysin of *T. forsythia* degrade these AMF and alter complement aspects of innate immunity [70]. Additionally, through its interaction with complement, one could propose an important role due to the clear data implicating complement activation and disease associated with *P. gingivals* [71–73].

Clear data demonstrate that exposure of gingival epithelial cells to oral bacteria that have been associated with periodontitis results in the production of an array of innate immune molecules, including β-defensins and CAMP (LL-37) [2]. Furthermore, the importance of the array of AMFs have been supported by a number of genetic disorders associated with decreased AMF production in the oral cavity, and increased susceptibility to bacterial infections [2, 74, 75]. Additional examples include mucin-7 and lactoferrin being significantly altered in patients with periodontitis [76]. Thus, we evaluated the relationship of this array of AMFs with expression of genes that would reflect a changing periodontal environment that would contribute to tissue alterations consistent with a disease process. The results showed few correlations between the AMF levels and the expression of the various tissue and environmental genes in the older group of animals. However, numerous significant correlations were observed in the younger group that were most noted in disease samples, and displayed generally positive correlations with inflammatory genes and significant negative correlations with the bone biology, tissue destructive/remodeling, and hypoxia genes. The numerous correlations were unexpected, but may reflect a more regulated response capacity of the gingival tissues in the younger group, which is consistent with less destructive disease [42].

It is also clear that the characteristics of the oral microbiome in the subgingival sulcus that is juxtaposed to the gingival tissues shows interactions that are critical for homeostasis. However, alterations toward a dysbiotic microbiome occur with disease and are reflected by substantial gene expression changes in the tissues. We have previously described the microbiome at sites of periodontal lesions in the non-human primates and how these differ in healthy and resolved lesion samples [33, 77–79]. The microbiome data presented distinct differences in samples from younger and older animals, as well as changes in the microbial distribution with disease in the younger group of animals. Interestingly, at the level of the 26 dominant microbial genera, the changes were more subtle in samples from the older animals with *Fretibacterium* the most distinctive of the changes. This relatively recently identified genera within the phylum Synergistetes has been identified in multiple studies as a potential microbial diagnostic marker of disease [80–82]. Moreover, it was identified as a member of a group of microbes that remained elevated in persistent aggressive disease patients post-therapy [83, 84]. Thus, the findings in this non-human primate model are reflective of the disease microbiome in humans.

The data was then interrogated to estimate relationships among specific microbial components of the microbiome and the patterns of AMF expression in the tissues. In both groups of animals, frequent significant correlations across a wide range of the AMF were observed in the healthy samples, with 20–25% greater frequency of positive correlations. In contrast, during disease ~40–50% fewer correlations were noted, although in both age groups the positive relationships dominated. Finally at resolution, while the positive and negative correlation frequencies were similar in both the age groups, the striking feature was the lower number of AMF genes that contributed to these correlations. There is a general lack of comparable literature with which to compare these findings; however, one interpretation is that with tissue homeostasis these AMF represent a family of genes that are responsive to the oral microbiome and may help regulate homeostasis. During disease this relationship is substantially decreased potentially reflecting the dysbiosis and host dysregulation, or being driven by members comprising the new dysbiotic microbiome. An additional question was also addressed regarding the biologic features of healthy tissues and gingival tissues that represent resolved lesions. The data showed a clear difference in AMF expression in healthy compared to disease resolved tissues in both age groups, with the greatest loss of relationships in the resolution samples from the older animals.

A previous study focused on an array of antimicrobial peptides (AMPs) that showed a wide variation between individuals and were modulated by disease [50]. The results suggested salivary AMPs present in periodontal health and disease showed some correlations of specific antimicrobial peptides and clusters of these AMPs with bacterial species and clusters of species in the oral microbiome. Our results drilled down to individual OTU/species level and explored these relationships with the broader AMF gene levels in health, disease and resolution. In health, the portfolio of individual bacteria showed 54 and 73% with elevated correlations in the younger and older groups, respectively. The microbial representation of the correlations with AMF was decreased greatly during disease, with only 4 OTUs in the younger group and only 30% of the bacteria in the older group samples, although these were comprised of *Fusobacterium, Bacteroidetes, Porphyromonas, Prevotella, Treponema, Fretibacterium*, and *Chloroflexi* genera. Similarly, a substantial difference in the correlation patterns between the bacteria and AMF was seen in the younger vs. the older animal resolution samples. The samples from the younger animals appeared to re-establish the AMF-bacterial correlations in resolution samples, while these were totally lost in the older animal specimens.

Finally, as periodontitis reflects changes in the geospatial and functional aspects of microbial complexes in the subgingival microbiome [85–88] we explored the presence of microbial consortia and estimated their combined role in regulating the AMF patterns with age and in health and disease. We had previously reported on these types of microbial consortia that were significantly associated with gingival tissue responses of apoptosis, autophagy and hypoxia genes [77, 78]. Additionally, we identified other microbial complexes in the microbiome of young and older animals that related to the expression of an array of immunoglobulin genes in the gingival tissues (in press). Thus, we identified multiple microbial complexes in the young and older animal samples that significantly correlated with a group of the AMF in health and disease. The features of these complexes identified clearly different complexes in the young and older animal samples in both health and disease. Individual complexes in the young animals showed a skewing toward either positive or negative correlations with the AMF in health or disease. In contrast, there was a distinct predilection for individual complexes to exhibit positive or negative correlation with the array of AMF. The complexes also demonstrated a prevalence of more pathogenic species, mostly commensals, or a mixture of these species within the same complex. Finally, there was generally limited overlap in the specific AMF that correlated with these complexes within health or disease samples in either age grouping. These results supported, as was observed with the relationship to apoptosis, autophagy, hypoxia and immunoglobulin genes, somewhat distinct patterns of microbial complex relationships to these host AMF responses, which underpins certain host-microbe interactions that may be critical for the maintenance of gingival homeostasis or can reflect a basis for the transition to a disease process. Further work remains to better understand the detailed temporal nature of these interactions and potential utilization for these parameters for estimating risk of disease progression or more limited response to standard therapy.

## Data Availability Statement

The datasets presented in this study can be found in online repositories. The names of the repository/repositories and accession number(s) can be found below: https://www.ncbi.nlm.nih.gov/, GSE180588; https://www.ncbi.nlm.nih.gov/, PRJNA516659.

## Ethics Statement

The protocol was approved by the Institutional Animal Care and Use Committees (IACUC) of the University of Puerto Rico and University of Kentucky and a ligature disease model was utilized.

## Author Contributions

JE and OG developed and implemented the experimental design, obtained samples, interpreted the results, and constructed the report. SK provided technical assistance in preparation and analysis of the samples, provided content, and reviewed the manuscript. LN provided the advanced statistical analyses on the data, provided content, and reviewed the manuscript All authors contributed to the article and approved the submitted version.

## Funding

This work was supported by National Institute of Health grant P20GM103538.

## Conflict of Interest

The authors declare that the research was conducted in the absence of any commercial or financial relationships that could be construed as a potential conflict of interest.

## Publisher's Note

All claims expressed in this article are solely those of the authors and do not necessarily represent those of their affiliated organizations, or those of the publisher, the editors and the reviewers. Any product that may be evaluated in this article, or claim that may be made by its manufacturer, is not guaranteed or endorsed by the publisher.

## Supplementary Material

Supplementary Figure 1 **(A–D)** Altered transcript levels expressed as fold-difference from baseline healthy levels to initiation (0.5 months), progression (1 and 3 months) and resolution (5 months) of periodontal lesions in each age group.

Supplementary Figure 2 Differences in AMF expression levels based upon categorization of bacterial families in the microbiome into high and low levels. Each point denotes the value of an AMF gene level based upon expression in high vs. low family microbiome samples.
