# Supplementary figures and images for: Gingival Transcriptome of Innate Antimicrobial Factors and the Oral Microbiome With Aging and Periodontitis
Source: Front Oral Health. 2022 Mar 7;3:817249. doi: 10.3389/froh.2022.817249 (PMC8940521; doi:10.3389/froh.2022.817249)

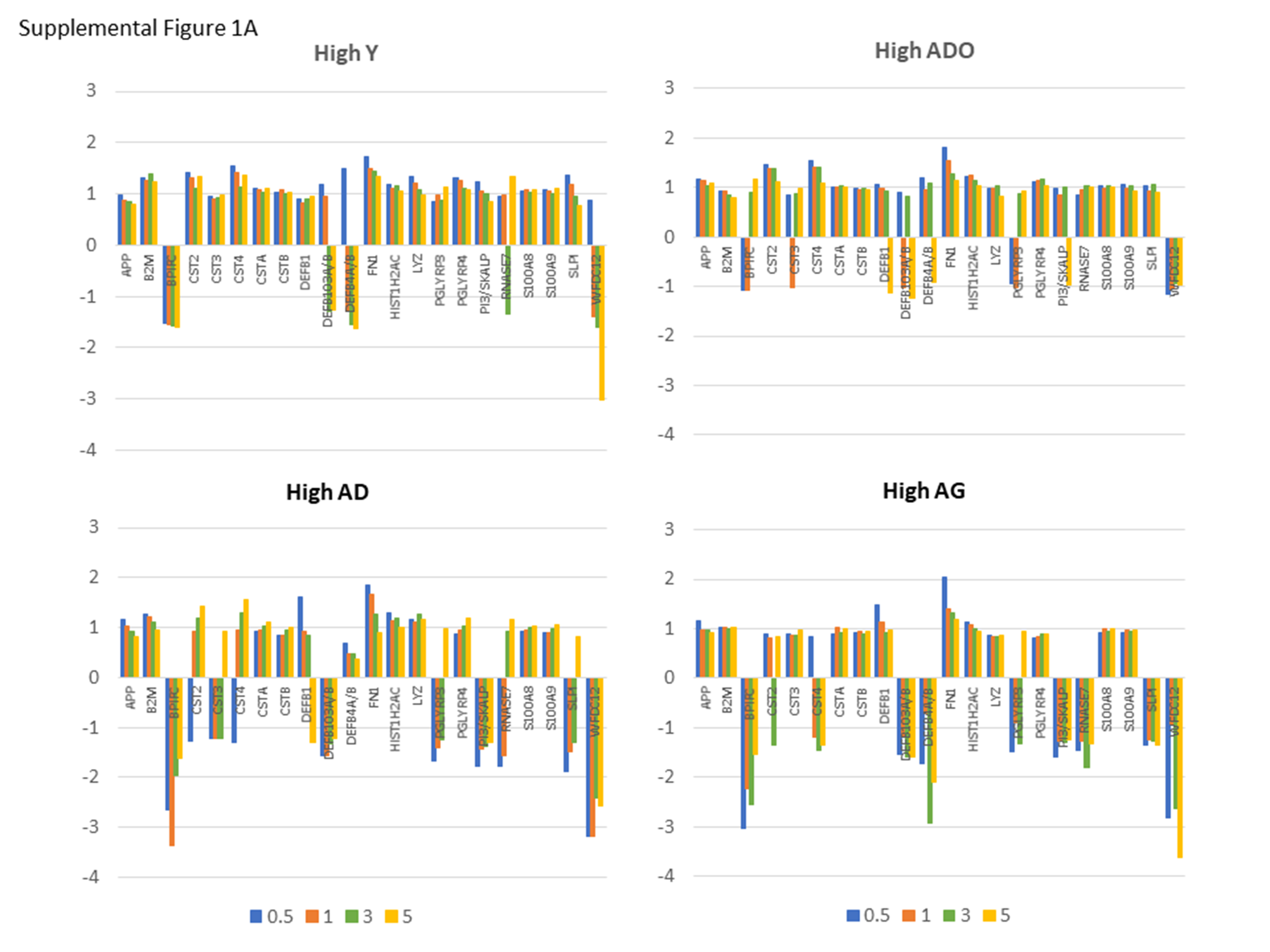

Supplement: Supplementary Figure 1 — (A–D) Altered transcript levels expressed as fold-difference from baseline healthy levels to initiation (0.5 months), progression (1 and 3 months) and resolution (5 months) of periodontal lesions in each age group. [file Image_1.tif]

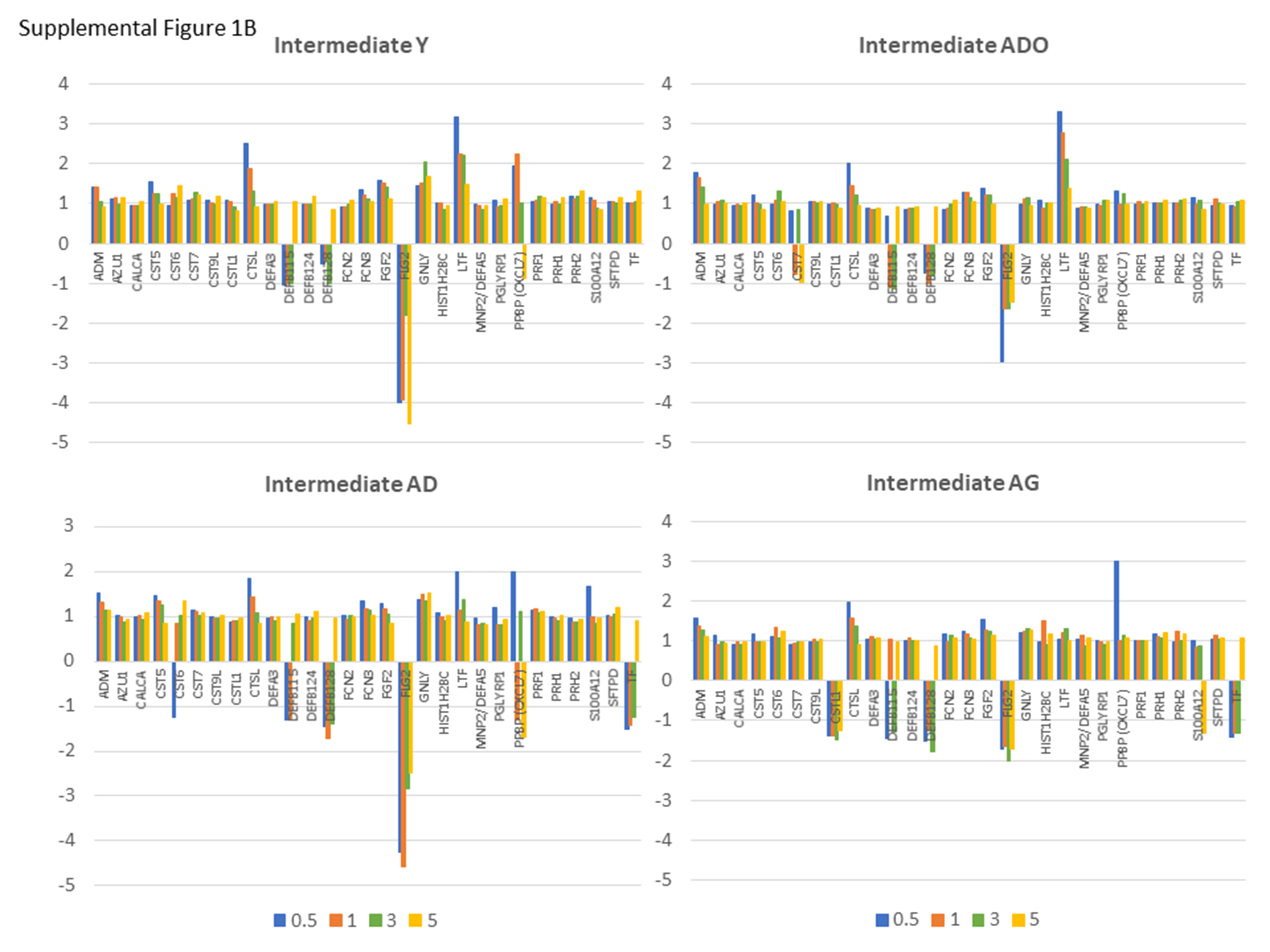

Supplement: Supplementary file 2 [file Image_2.tif]

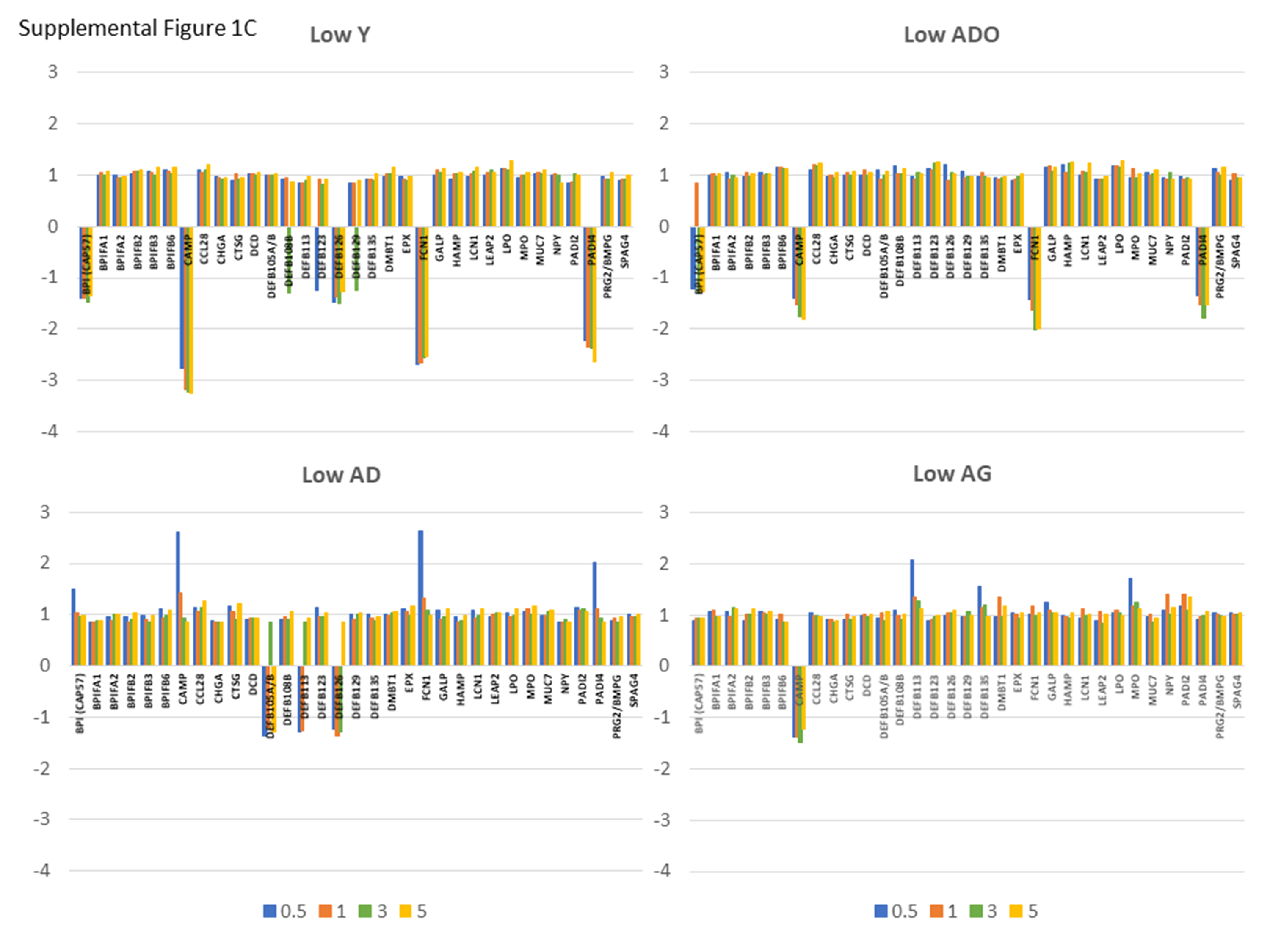

Supplement: Supplementary file 3 [file Image_3.tif]

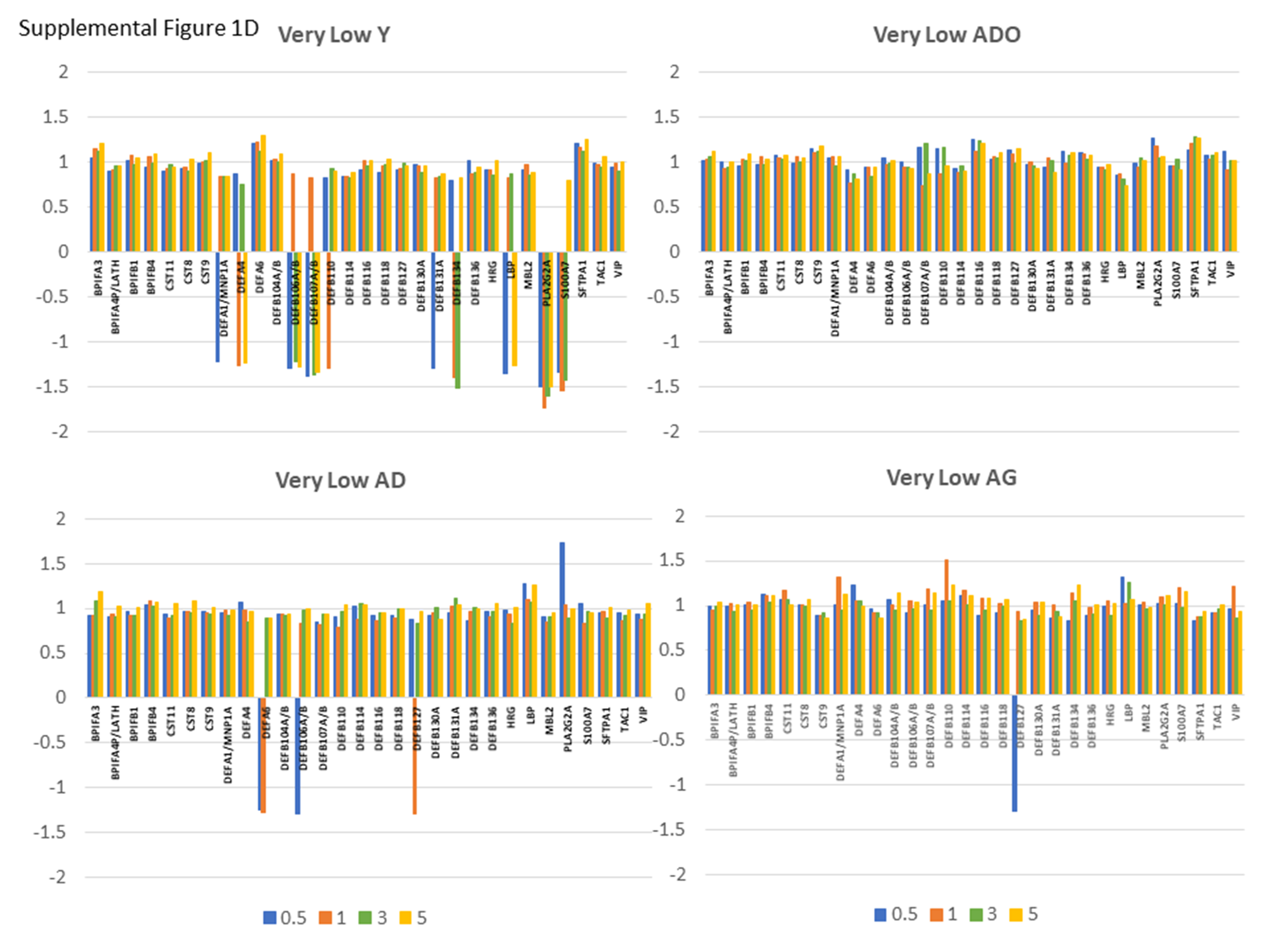

Supplement: Supplementary file 4 [file Image_4.tif]

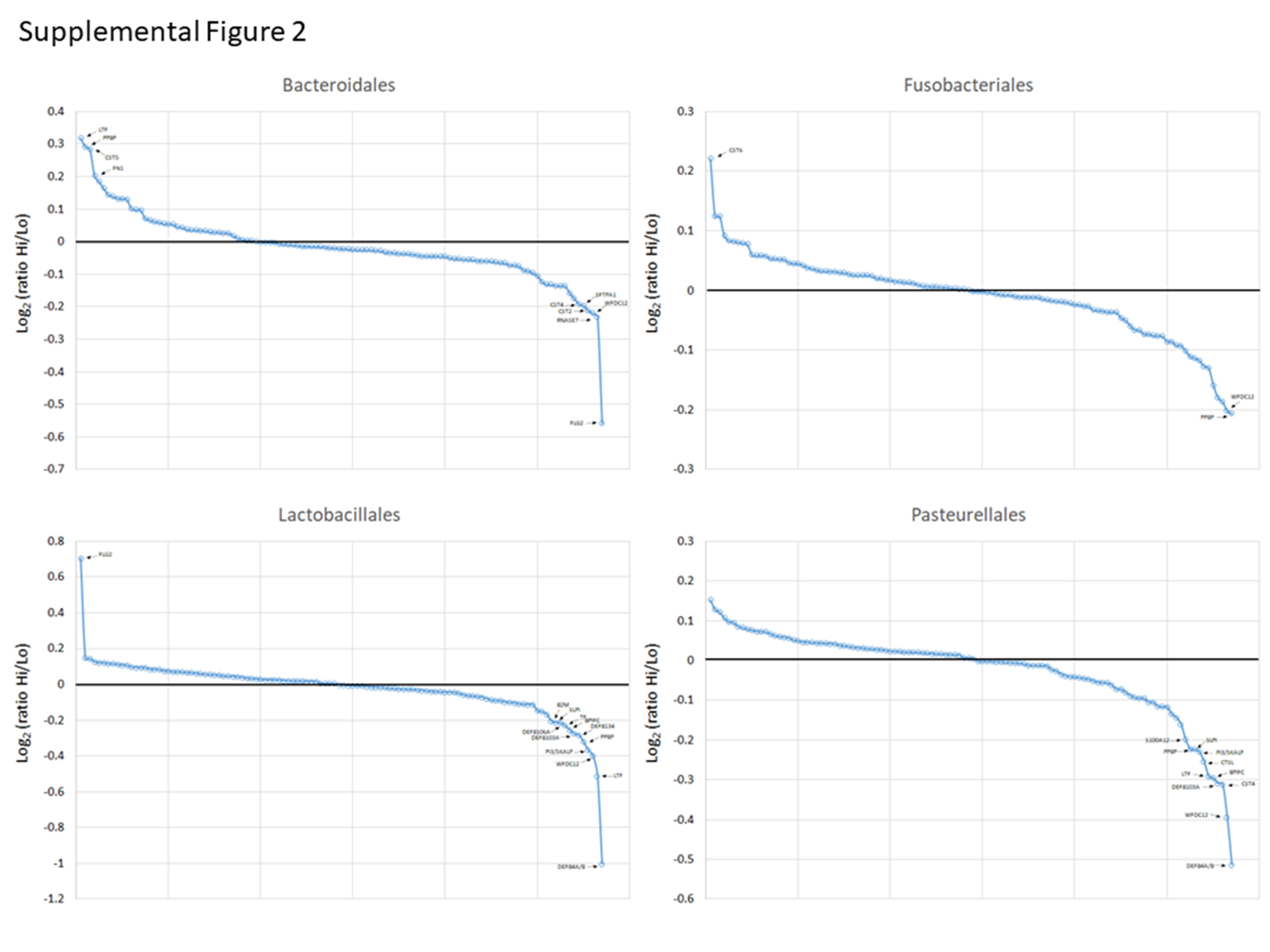

Supplement: Supplementary Figure 2 — Differences in AMF expression levels based upon categorization of bacterial families in the microbiome into high and low levels. Each point denotes the value of an AMF gene level based upon expression in high vs. low family microbiome samples. [file Image_5.tif]

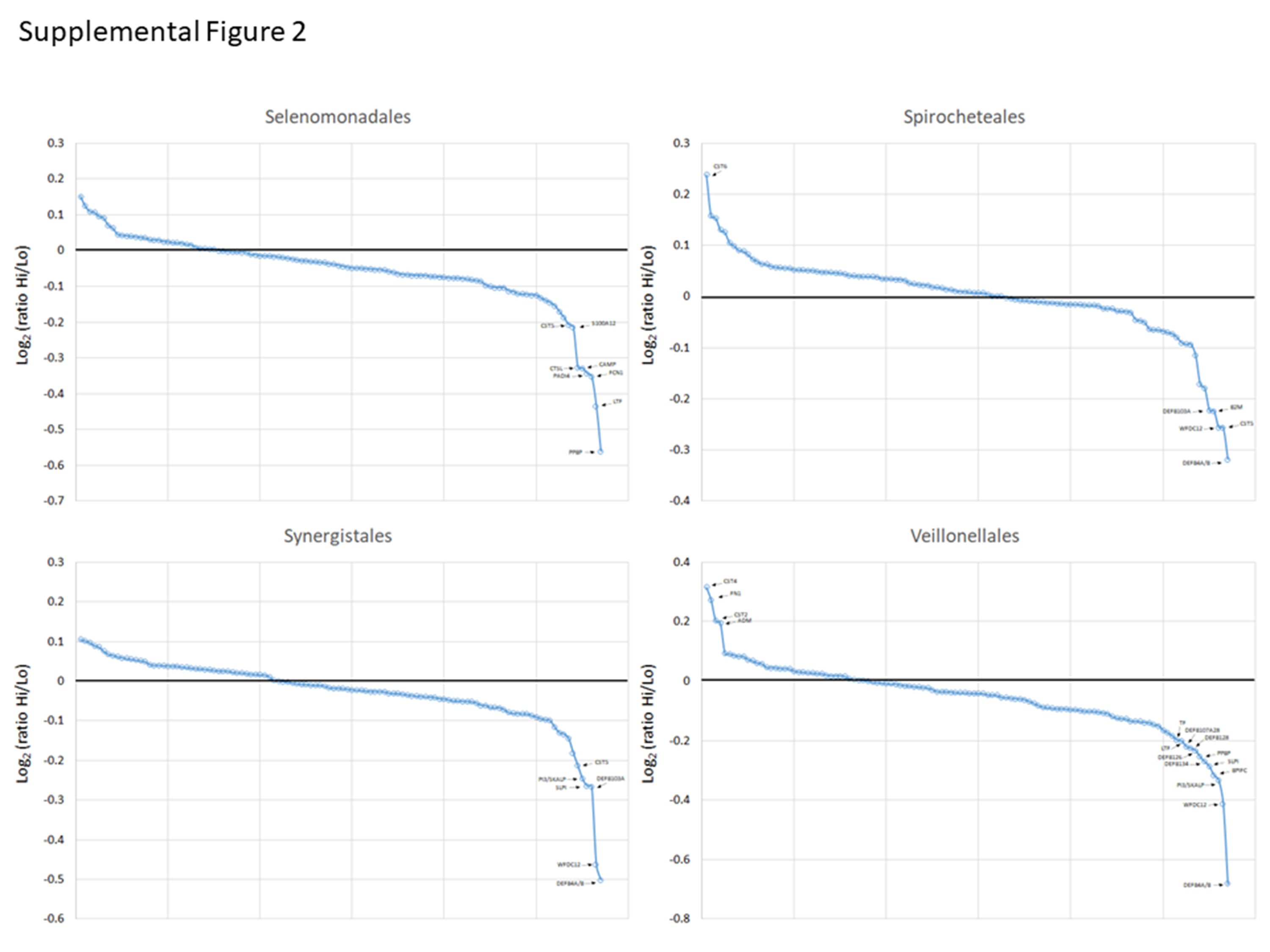

Supplement: Supplementary file 6 [file Image_6.tif]
